# Advances in Flame Retardant Technologies for Epoxy Resins: Chemical Grafting onto Carbon Fiber Techniques, Reactive Agents, and Applications in Composite Materials

**DOI:** 10.3390/molecules29245996

**Published:** 2024-12-19

**Authors:** Omar Dagdag, Hansang Kim

**Affiliations:** Department of Mechanical Engineering, Gachon University, Seongnam 13120, Republic of Korea; omardagdag@gachon.ac.kr

**Keywords:** epoxy resin, hexachlorocyclotriphosphazene, grafting, carbon fiber, interfacial bonding, properties, composite materials

## Abstract

This review examines recent advancements in flame retardant technologies for epoxy and bio-epoxy resins, focusing on the chemical grafting of hexachlorocyclotriphosphazene (HCCP) onto carbon fibers (CFs) and the use of reactive flame retardant agents in composite materials. It covers various grafting techniques and analyzes mechanisms behind property enhancements, exploring how these approaches improve thermal stability and flame resistance while addressing sustainability challenges. This review discusses the synergistic effects of bio-based materials and innovative grafting methods. It presents applications of flame-retardant epoxy–carbon fiber composites, demonstrating the potential of HCCP-CF composites as high-performance, environmentally friendly structural materials. Additionally, this review evaluates the impact on mechanical properties and interfacial bonding, crucial aspects often affected by flame retardant treatments. This comprehensive overview provides valuable insights, identifying current challenges, future directions, and potential breakthroughs in the field of advanced composite materials.

## 1. Introduction

**Scheme 1:**
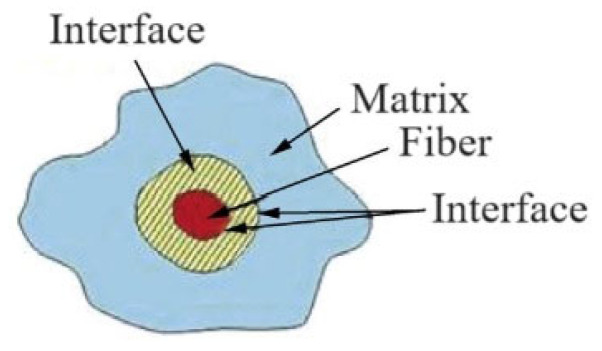
Schematic representation of the interface structure of CF/polymer matrix composites.

Composite materials are made up of two or more immiscible elements, whose combination provides properties superior to those of the individual components [1,2,3,4]. Composite products have two elements: the matrix and reinforcement, and the 3D region with distinct properties between these two components is known as the interphase region. In contrast, the interface, with its 2D structure, serves as the barrier between the constituents (Scheme 1). Generally, a matrix (often a polymer) encapsulates and holds in place reinforcements (carbon and glass fibers, etc.), providing strength and mechanical rigidity [5,6,7].

Composite materials offer many advantages, such as an excellent strength-to-weight ratio, good fatigue and corrosion resistance, and great design flexibility [8,9]. They are widely used in a variety of industries, including in aerospace, automotives, construction, and sports, due to their ability to meet specific performance and design requirements [10,11].

CF are widely used as reinforcements in composites, where they are combined with polymer matrices to form CFRP materials [12,13]. CFs are extremely fine fibers composed mainly of carbon atoms. They are produced by heating organic precursor fibers, such as PAN fibers, to high temperatures in an inert environment [14,15,16]. This process eliminates the non-carbon elements, leaving behind a highly ordered and resistant carbon structure [17,18]. They possess excellent electrical and flexibility properties, as well as temperature resistance, compressive strength, high tensile strength, low density, thermal conductivity, and chemical inertness [19,20,21]. CFs are in high demand in a wide range of industries, including civil engineering, aerospace, and automobiles [22,23,24]. CFs are commonly combined with other materials, such as epoxy resins (EPs), to create composite materials that have a high strength-to-weight ratio. Despite their relatively high manufacturing costs, CFs are increasingly being used due to their unique performance characteristics [25,26].

HCCP is an intriguing chemical compound that is increasingly recognized in various research and industrial sectors due to its distinctive properties and broad applicability [27]. This organophosphorus compound features a remarkable molecular structure, consisting of six chlorine atoms attached to a central phosphorus (P) and nitrogen (N) ring [28]. Its robust structure and chemical reactivity position it as an excellent candidate for the synthesis of polymers, organometallic compounds, and functional inorganic materials [29]. One of HCCP’s most compelling characteristics is its capacity to polymerize under diverse conditions, resulting in organophosphorus polymers that exhibit outstanding properties such as flame resistance, thermal stability, and biocompatibility [30,31]. These polymers find applications in a variety of fields, including selective membranes, flame retardant coatings, and biomedical uses [32,33]. Additionally, HCCP is extensively utilized as a crosslinking or surface modification agent across multiple industries, including catalysis, electronics, and materials chemistry [34,35]. Its ability to functionalize surfaces and enhance intermolecular interactions makes it a valuable tool for improving the performance of composites, adhesives, and coatings [36,37,38].

Grafting HCCP onto CFRP represents an innovative strategy in polymer chemistry and composite development aimed at enhancing material properties [39]. This grafting technique entails the controlled application of HCCP to the surfaces of carbon fiber composites. HCCP is a cyclic inorganic compound that contains phosphorus and nitrogen atoms [40,41]. When grafted onto CFs, it forms chemical bonds that modify the surface characteristics of the composites. The primary objective of this approach is to enhance the interfacial properties between the CFs and the polymer matrix, which can result in improved adhesion, resistance to mechanical stress, and resilience against chemical degradation. By strengthening the bonds between CFs and the polymer matrix, grafting HCCP can enhance several properties, including improved adhesion, reinforced interfaces, enhanced mechanical performance of the composite materials, increased fire resistance, greater thermal stability of CFRP, reduced smoke emissions, and functionalization of CF surfaces that allows for further bonding with other materials or chemical modifications. These methods create a uniform layer of HCCP on the CF surfaces, resulting in a fortified interface with the polymer matrix. Ongoing research is focused on refining grafting techniques, exploring new possibilities, and developing HCCP-grafted CF materials with even superior properties.

This review discusses recent advancements in flame retardant technologies for EPs, particularly emphasizing the chemical grafting of HCCP onto CFs and the application of reactive flame retardants. It examines the synthesis methods, properties, and applications of HCCP-grafted CF composites, underscoring their potential to enhance safety, performance, and sustainability across various fields. Additionally, this study explores future prospects for this emerging class of materials within composite technology.

## 2. Chemical Grafting onto Carbon Fiber Techniques

### 2.1. HCCP Grafting onto CF Composites

HCCP and CF are two essential materials, each possessing unique properties that render them valuable across various applications. HCCP is a cyclic inorganic compound characterized by its high phosphorus content and exceptional flame retardant capabilities, while CF is a lightweight, high-strength material known for its excellent electrical and thermal conductivity. The combination of HCCP and CF in composite materials can create a synergistic effect that enhances overall performance. Research has shown that HCCP-CF composites exhibit improvements in thermal stability, electrical conductivity, mechanical properties, and flame retardancy. These advanced materials hold promise for applications in diverse sectors, including automotives, energy storage, aerospace, and electronics.

HCCP-CF composites are produced by grafting HCCP onto the surface of CFs. The optimal preparation method varies based on the specific properties and performance characteristics desired in the final composite.

Zhang et al. [42], used in situ polycondensation of 4,4′-diamino diphenyl ether (DDE) and HCCP to produce amine-capped cross-linked polyphosphazene (APC) grafted onto CF. This method improves the interfacial properties of the fibers and matrix, thereby increasing the effectiveness of carbon fiber-reinforced composites.

Grafting HCCP onto CFs improves their mechanical properties by increasing interfacial adhesion between the fibers and the polymer matrix. The process increases the density of reactive amine groups on the fiber surface, which can form strong chemical bonds with functional groups in the matrix, such as those found in M-PP. This strong bonding reduces the likelihood of fiber pull-out during mechanical loading, resulting in better stress transfer and load-bearing capacity. Furthermore, the uniform coating of polyphosphazenes helps to distribute mechanical stress evenly across the fiber surface, reducing localized stress concentrations that can cause failure. As a result, composites reinforced with HCCP-grafted carbon fibers have higher tensile strength, stiffness, and overall durability than those made with untreated fibers.

**Figure 1:**
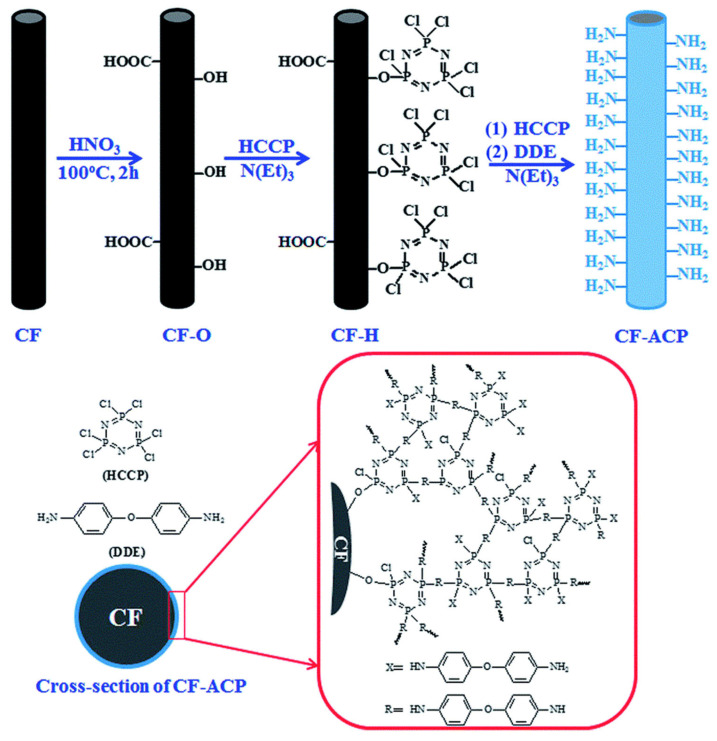
A diagram depicting the grafting procedure for amino-capped cross-linked HCCP-CF [42]. Copyright 2014 RSC.

Figure 1 clearly illustrates the various synthesis techniques and processes, while the different synthesis steps are presented in Table 1.

The study found that CF-ACP/M-PP composites had a significantly IFSS of 32.3 MPa, representing a 223.0% increase over unmodified CF/M-PP composites, which had an IFSS of only 10.0 MPa (Table 2). This improvement is due to the successful grafting of ACP onto CFs, which increases wettability and promotes stronger chemical bonding with the maleic anhydride-grafted polypropylene (M-PP) matrix. These findings show that the ACP modification improves the interfacial properties and overall mechanical performance of the composites.

Luo et al. [43] developed a novel 3D reinforcement by grafting epoxy-functionalized graphene oxide (EG) onto CF.

**Figure 2:**
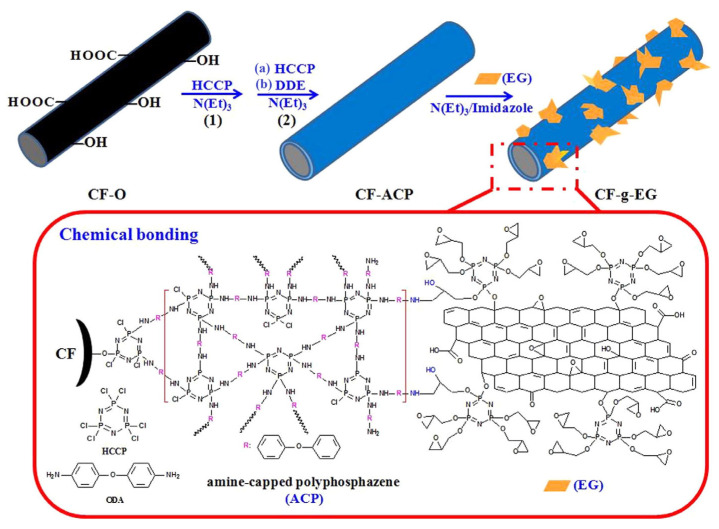
A schematic of the CF-g-EG grafting methods [43]. Copyright 2017 Elsevier.

Figure 2 shows a schematic diagram of the CF-g-EG grafting methods, while the different synthesis steps are presented in Table 1. The study found that EG onto CF-ACP improves the interfacial properties of CF reinforced composites. According to the study, the composites’ IFSS increased from 47.3 MPa for virgin carbon fiber composites to 97.7 MPa for functionalized composites, representing a 106.5% improvement (Table 2). This improvement is due to the formation of chemical bonds and strong mechanical interlocking between the CFs and the EP matrix, which is aided by the grafted EG sheets’ unique petal-like structure, which increases specific surface area and promotes better wettability with the epoxy resin.

**Figure 3:**
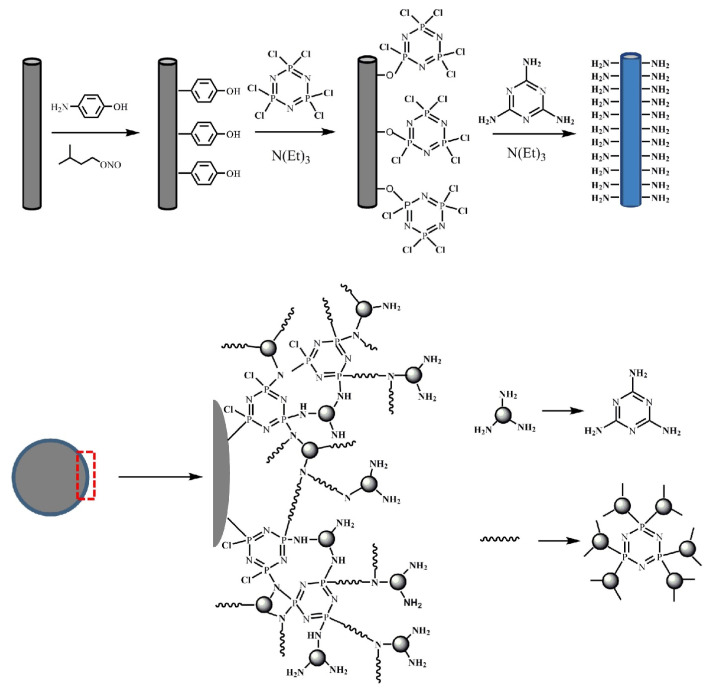
Schematic of the grafting method [44]. Copyright 2017 Elsevier.

Li et al. [44], employing simple in situ polymerization, prepared an amino-terminated nitrogen-rich layer of poly(cyclotriphosphazene-co-melamine) (PPM) coating to functionalize CF and improve the interfacial adhesion of CF-reinforced PPBES. The grafting method is presented in Figure 3.

**Figure 4:**
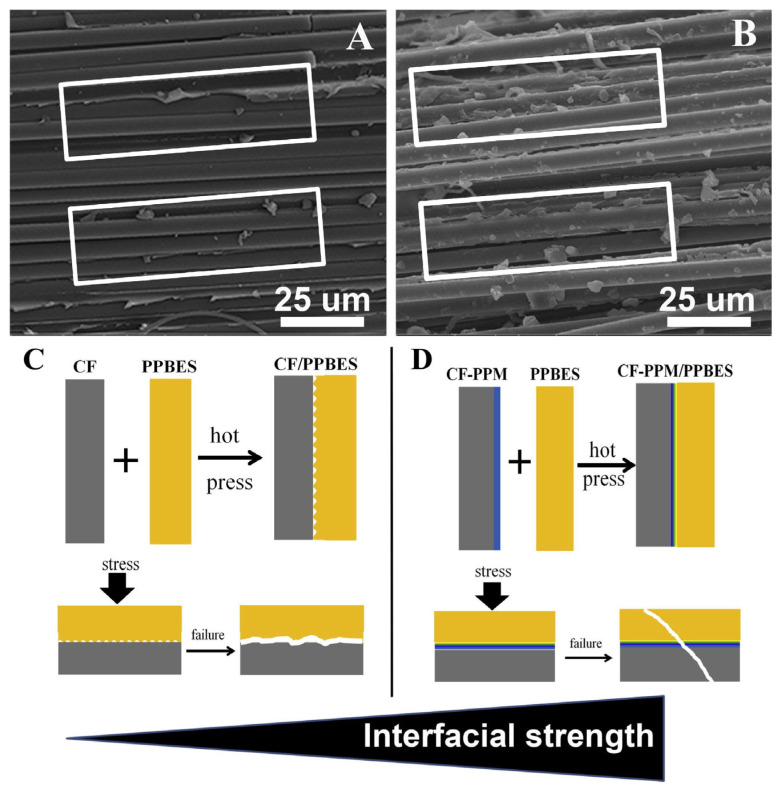
SEM patterns of the ILSS fracture surface and the proposed composite failure mechanism of (**A**,**C**) CF/PPBES and (**B**,**D**) CF-PPM/PPBES [44]. Copyright 2017 Elsevier.

CF-reinforced copoly(phthalazinone ether sulfone) (PPBES) composites were made in several steps. To remove polymer sizing and contaminants, bare CFs were first refluxed in acetone for 24 h. The cleaned CFs were then treated with 4-aminophenol and isopentyl nitrite in DMF to introduce hydroxyl groups, producing CF-OH. Following that, CF-OH was immersed in a DMSO solution containing HCCP and triethylamine, and then melamine (MA) was added to promote the formation of a nitrogen-rich PPM coating on the CF surface, yielding CF-PPM. Unreacted monomers and impurities were removed from the modified fibers by washing them. Finally, the CF-PPBES composites were created by immersing CF tows in a PPBES/DMAc solution, followed by solvent evaporation and hot pressing to achieve the desired composite structure. CF reinforced PPBES composites’ interfacial properties improved significantly as a result of surface modifications to the CFs (Table 1). The composites’ ILSS increased significantly, with CF-OH composites achieving an ILSS of 89.2 MPa, representing a 14.9% improvement over bare CF composites with an ILSS of 77.6 MPa. More impressively, the ILSS for CF-PPM composites reached 95.6 MPa, a significant 23.2% improvement (Table 2). This improvement in interfacial adhesion was attributed to chemical bonding and increased wettability facilitated by the nitrogen-rich PPM coating, which also contributed to a shift in the failure mechanism from interface failure in bare CF/PPBES composites to matrix failure and fiber breakage in CF-PPM/PPBES composites, indicating a stronger and more effective interface. PPM coatings significantly improve the mechanical properties of CF reinforced PPBES composites. Specifically, the flexural strength of CF-PPM composites increased to 2067 MPa, a 29.3% increase over the 1598 MPa observed in uncoated CF/PPBES composites. Similarly, the flexural modulus increased significantly, reaching 156.1 GPa, representing a 31.0% increase over the counterpart composites. These enhancements are primarily due to the PPM coating’s increased interfacial adhesion between the CFs and the PPBES matrix, which allows for better stress transfer during mechanical loading. The coating improves the surface wettability and roughness of the CFs, resulting in more efficient load transfer and lower stress concentration at the interface. Furthermore, the PPM coating promotes stronger hydrogen bonding and intermolecular forces between the fibers and matrix, which improves the overall mechanical performance of the composites. SEM analysis of CF/PPBES composites’ fractured surfaces (A) revealed significant interface failure, as evidenced by numerous exposed bald CFs and visible interface debonding, indicating poor fiber-to-matrix adhesion (Figure 4A). In contrast, the CF-PPM/PPBES composites demonstrated a compact and clean fracture section, with no evidence of interface debonding or fiber extraction from the matrix, indicating strong interfacial adhesion (Figure 4B). The analysis reveals that the composite interface contains numerous structural defects, which can lead to stress concentration and rapid crack propagation under stress, resulting in catastrophic interface failure (Figure 4C). However, the chemical grafting of a PPM layer onto the CF surface improves the surface energy and roughness, enhancing the resin’s ability to wet the fibers. This modification introduces nitrogen, phosphorus heteroatoms, and amino groups that improve compatibility and strengthen intermolecular forces between CF-PPM and PPBES. In contrast, fewer defects are observed in the modified interface, allowing for effective load transfer from the matrix to the fibers, which helps prevent crack initiation (Figure 4D). The PPM layer also mitigates crack propagation by passivating crack tips and promoting crack diversion. Consequently, the failure of CF-PPM composites occurs through fiber breakage and resin yielding, demonstrating strong interfacial bonding that aligns with the observed improvements in ILSS and flexural strength. 

**Figure 5:**
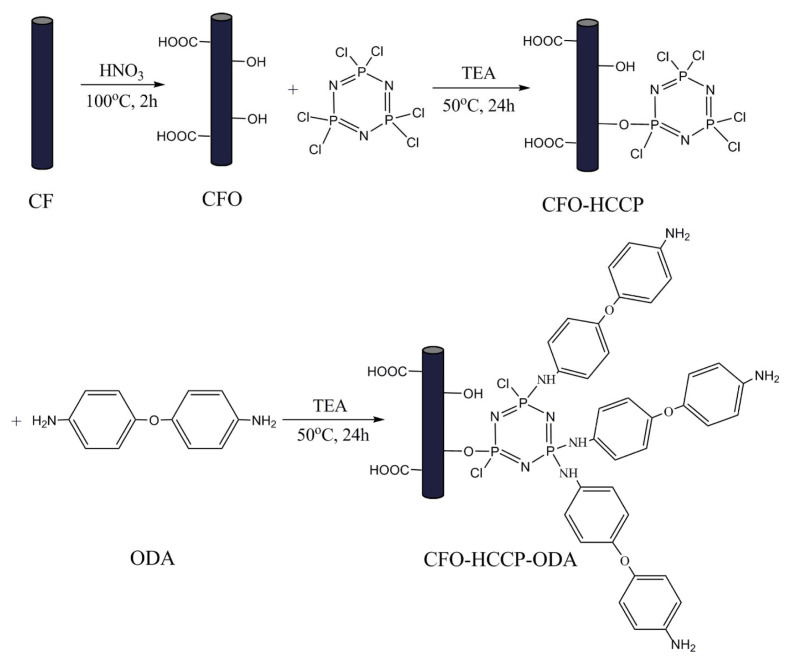
A schematic of the CFO-HCCP-ODA grafting methods [39]. Copyright 2014 Elsevier.

Xu et al. [39] created a highly effective approach for putting reactive amines onto CF surfaces by employing HCCP as a novel coupling agent. The grafting method is presented in Figure 5.

The main findings on the interfacial properties of CF reinforced composites show that functionalizing carbon fibers significantly improves IFSS. Specifically, the IFSS values for desized CFs, CFO, and those modified with HCCP and ODA (CFO-HCCP-ODA) were 47.5 MPa, 56.6 MPa, and 81.3 MPa (Table 2). This corresponds to a 19.2% increase in IFSS for CFO compared to CF, and a remarkable 71.2% increase for CFO-HCCP-ODA compared to desized CF. Furthermore, the tensile strength of a single CF is 4.80 GPa, CFO is 4.69 GPa, and CFO-HCCP-ODA is 4.75 GPa. The findings revealed that the tensile strength of CFs decreased marginally after functionalization, with CFO losing 2.3% and CFO-HCCP-ODA losing 1.0% when compared to CF. However, CFO-HCCP-ODA had a 1.3% higher tensile strength than CFO, indicating that mechanical properties were retained after functionalization.

**Figure 6:**
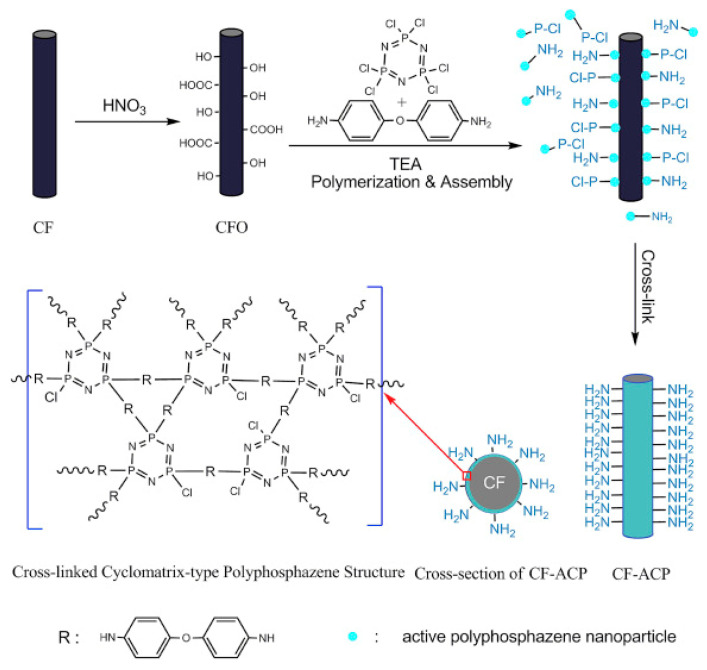
Surface functionalization of CF using polyphosphazene coating [49]. Copyright 2016 Elsevier.

A further study by the same group [49] demonstrated a straightforward, efficient, and alternative surface treatment technique for functionalizing CF surfaces with a large number of active amines by grafting 4,4′-oxydianiline (ODA), using HCCP as a new coupling agent, and operating under mild reaction conditions. Figure 6 illustrates a schematic of grafting processes.

Grafting HCCP onto CFs improves their mechanical properties by adding a strong polyphosphazene coating that improves interfacial adhesion with the epoxy matrix. The active chlorinated groups in HCCP facilitate nucleophilic substitution reactions with amines, resulting in the formation of a crosslinked network that fills surface defects while also forming strong chemical bonds between the fiber and the matrix. This improved bonding causes better load transfer during mechanical stress, increasing the overall tensile strength and durability of CF-reinforced composites. Furthermore, the polyphosphazene coating absorbs and dissipates stress, lowering the risk of fiber fracture and contributing to a more resilient composite structure.

The study found that the addition of CF-ACP significantly enhanced the interfacial and mechanical properties of CF/EP composites. The IFSS of the modified composites increased by an impressive 70.5% compared to raw CF composites, indicating a substantial improvement in adhesion between the CFs and the EP matrix. This enhancement is primarily attributed to increased surface wettability and the formation of strong chemical bonds facilitated by the amine groups in the polyphosphazene coating. Additionally, the coating effectively repairs surface defects on the CFs, further improving interfacial performance. The tensile strength of the coated fibers also saw a significant increase, reaching 5.31 GPa, which represents increases of 10.6% over uncoated CFs and 13.2% over CFO. This rise in tensile strength is linked to the coating’s ability to heal surface defects and enhance the structural integrity of the fibers.

**Figure 7:**
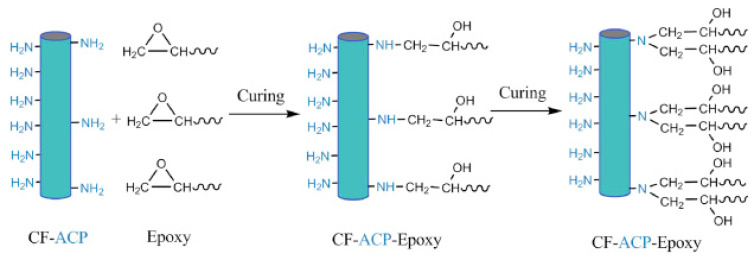
The diagram depicts the interfacial response between CF-ACP and EP matrix [49]. Copyright 2016 Elsevier.

Figure 7 illustrates that the cyclomatrix-type polyphosphazene layer on CFs enhances chemical interactions with the EP matrix, leading to improved interfacial adhesion and performance of CF/EP composites. The presence of amine groups increases surface energy, enhancing the wettability of CFs and EPs. Active amines can also react with EP groups during the curing process, resulting in significant chemical interactions that further strengthen the bond between CFs and the EP matrix. Overall, applying a cyclomatrix-type polyphosphazene coating not only enhances interfacial properties but also increases the tensile strength of the fibers, making it a promising method for improving adhesion while maintaining mechanical performance in composite materials.

**Figure 8:**
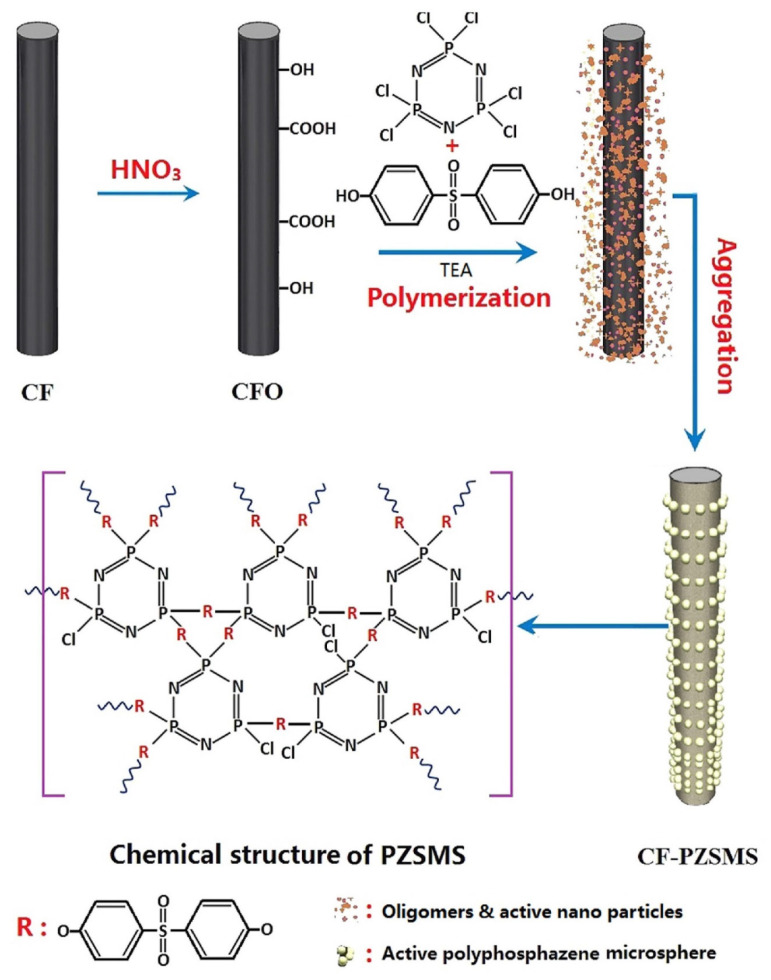
A schematic of the CF-PZSMS preparation process [50]. Copyright 2016 Elsevier.

Chen et al. [50] worked on creating a simple one-pot process for producing polyphosphazene microspheres (CF-PZSMS) hybrid to enhance the interfacial adhesion of CF-reinforced polymeric composites. The functionalization process is depicted in Figure 8.

**Figure 9:**
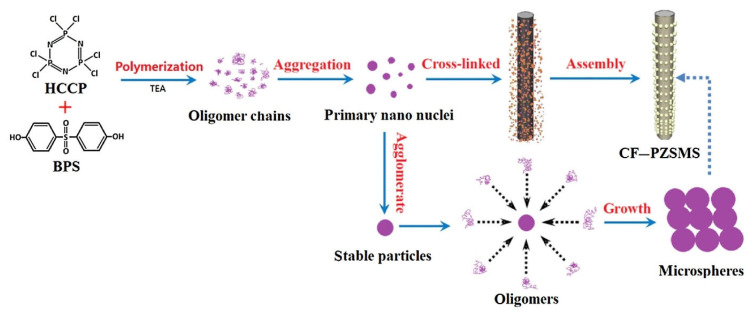
Possible process for the formation of CF-PZSMs [50]. Copyright 2016 Elsevier.

The polymerization process follows an oligomeric species absorption mechanism, as illustrated in Figure 9.

The mechanism of absorption by oligomeric species begins in the early stages of polymerization, when cyclomatrix-type polyphosphazene micro-nuclei are formed by the aggregation of oligomer chains caused by nucleophilic substitution (SN) between the hydroxyl (OH) groups of BPS and the P-Cl groups of HCCP. As these oligomers and primary nano nuclei with active P-Cl or OH groups bond to the surface of oxidized CFs, a polyphosphazene coating is formed by cross-linking. Concurrently, the polyphosphazene nano nuclei in solution aggregate into stable particles that absorb oligomers, eventually forming microspheres. The PZSMS are then immobilized on the CF surfaces by physical or chemical adsorption, in which the active groups (P-Cl, OH) react with one another. This immobilization technique increases the contact area between polyphosphazene microspheres and the CF surface, which improves the mechanical interaction of the fiber and matrix in polymer composites.

The interfacial property testing revealed a significant improvement in the IFSS of the CF/EP composites after the incorporation of polyphosphazene microspheres (CF-PZSMS), with a 22.98% increase compared to untreated CFs, resulting in an IFSS value of 84.75 MPa. Furthermore, the single fiber tensile tests revealed that the modified fibers’ tensile strength increased from 4.17 GPa for untreated fibers to 4.82 GPa for CF-PZSMS (Table 2). These findings suggest that surface modification not only improves interfacial adhesion between the fibers and the epoxy matrix, but also positively influences the mechanical properties of the fibers themselves, thereby improving the overall performance of the CF/EP composites.

**Figure 10:**
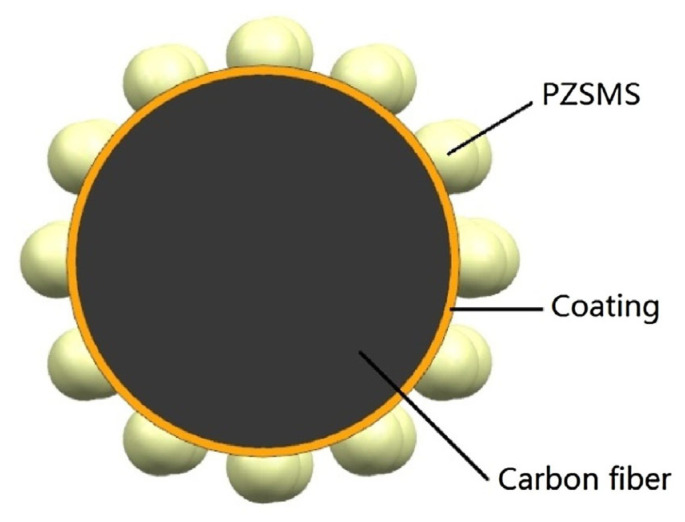
CF-PZSMS cross-sectional schematic [50]. Copyright 2016 Elsevier.

The CF-PZSMS cross-sectional schematic depicts the structural integration of PZSMS on the surface of CFs (Figure 10). This cross-section shows how the PZSMs are uniformly distributed around the CF, resulting in a hierarchical structure that improves interfacial adhesion between the fiber and the EP matrix. The polyphosphazene coating not only improves mechanical interlocking by increasing surface roughness, but it also contributes to the composite material’s overall tensile strength. This configuration improves stress transfer during loading, resulting in better performance characteristics for CF-PZSMS/epoxy composites compared to untreated fibers.

**Figure 11:**
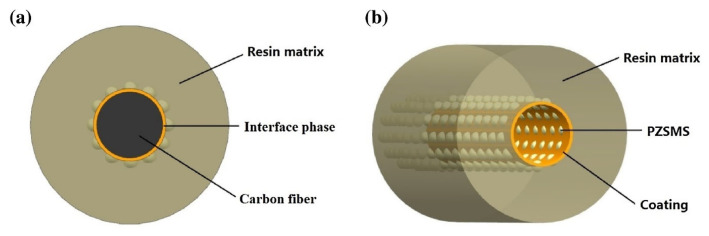
The interphase region of CF-PZSMS/EP composites is shown schematically in two views: (**a**) before debonding and (**b**) after [50]. Copyright 2016 Elsevier.

Figure 11 depicts a schematic illustration of the interphase area in CF-PZSMS/EP materials, emphasizing the interaction of the modified fibers with the EP matrix within the composite. In this interphase region, the incorporation of polyphosphazene microspheres produces a rough surface texture that improves the contact area, increasing the fracture surface area and limiting matrix sliding. Furthermore, these microspheres play an important role in efficiently transferring stress, preventing excessive stress distribution in areas of weakness and altering crack propagation pathways, resulting in improved mechanical performance of the composite material.

These findings demonstrate that the CF-PZSMS hybrid reinforcement enhances the interfacial adhesion and mechanical properties of CF-reinforced epoxy resin matrix composites.

**Figure 12:**
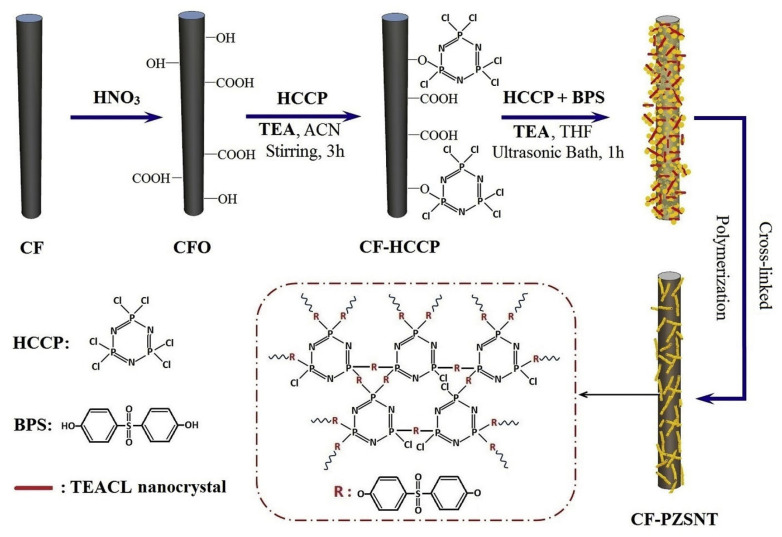
A schematic of the CF-PZSNT preparation process [51]. Copyright 2019 Elsevier.

A subsequent study by the same group [51] established a facile and effective technique for the fabrication of PZSNT/CF hierarchical hybrid reinforcement to increase CF surface roughness and energy, thus improving composite interfacial adhesion. The technique for preparing PZSNT/CF hierarchical hybrid reinforcement is presented in Figure 12.

**Figure 13:**
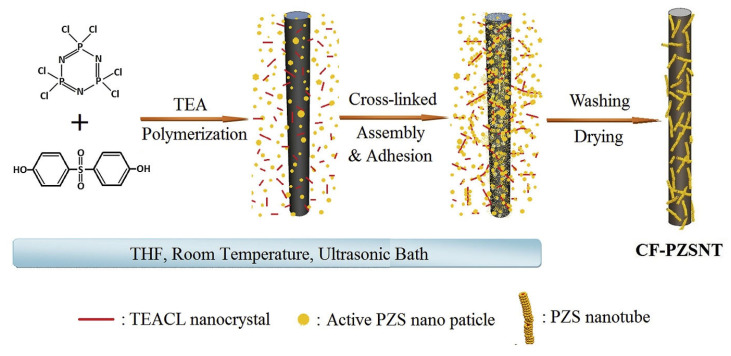
A schematic representation of the CF-PZSNT creation process [51]. Copyright 2019 Elsevier.

Figure 13 depicts a schematic diagram of the PZSNT/CF manufacturing process using an in situ template method. The most likely mechanism for the formation of PZSNT/CF is the polycondensation of BPS with HCCP via SN under ultrasonic irradiation, which results in the formation of PZS oligomers, primary PZS nanoparticles (NPs), and hydrochloric acid. TEA is used as an acid acceptor to neutralize HCl, forming TEACl and facilitating the polymerization process. Because of its low solubility in THF, TEACl precipitates and forms nanorod-shaped crystals that serve as nano-sized templates. When exposed to TEA, active PZS NPs adhere to the surface of TEACl and cross-link on the template surfaces, resulting in core/shell structures. This process causes PZS NPs to adhere to the surfaces of CF-HCCP fibers, forming a cross-linked PZS coating. Finally, the incorporation of PZS nanotubes into the PZS coating on the CF surface results in hierarchical PZSNT/CF reinforcements.

The key findings about the epoxy composites’ interfacial properties highlight a significant increase in IFSS, which increased from 68.91 MPa to 87.1 MPa after applying the polyphosphazene hybrid coating on CFs (Table 2). This improvement is attributed to increased surface roughness, better wettability, and stronger chemical bonding between the fibers and the epoxy matrix. The hierarchical structure of the PZS coating allows for effective mechanical interlocking, which improves stress transfer and reduces interfacial failure, ultimately leading to better mechanical performance of the composite. Furthermore, single fiber tensile strength tests revealed that, while the carbon fibers’ tensile strength decreased slightly (by 8.35%, from 4.55 to 4.17 GPa) due to surface oxidation during the modification process, the overall mechanical performance of the epoxy composites was significantly improved due to improved interfacial bonding, resulting in better energy absorption and crack resistance in the composite material.

**Figure 14:**
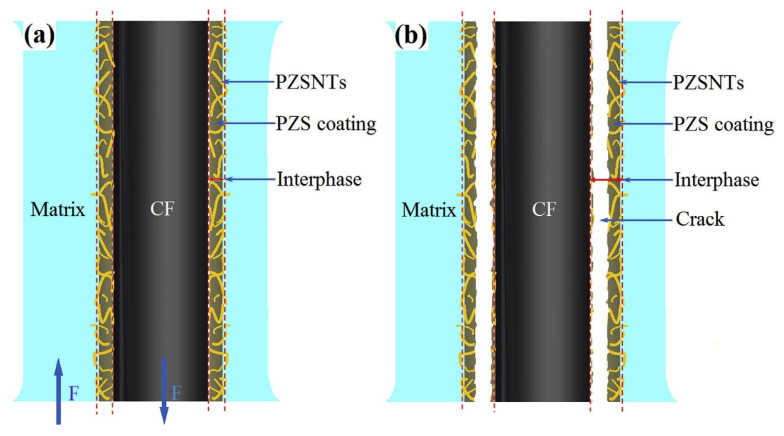
The interphase area of CF-PZSNT/EP composites is shown schematically in two views: (**a**) before debonding and (**b**) after [51]. Copyright 2019 Elsevier.

Figure 14 shows schematic depictions of the hierarchical interphase and interfacial characteristics of CF/epoxy composites with a polyphosphazene hybrid coating. In general, these findings show a significant improvement in interfacial characteristics, particularly IFSS, achieved by applying the polyphosphazene hybrid coating to CF surfaces. This study demonstrates the usefulness of a novel strategy for increasing the performance and mechanical behavior of CF/epoxy composites.

The same group discovered that PZSNTs grown on CFs using a remarkably simple polycondensation method can significantly increase the interfacial adhesion of CF/EP materials (Figure 12) [52]. This study developed PZSNTs/CF hierarchical hybrid improvements using optimization parameters and investigated the effect of CFs alteration on the interfacial interaction of CF-reinforced PA6 (CFs/PA6) materials. The composites’ interfacial property results and interfacial microstructure analysis revealed a significant improvement in IFSS after applying the PZSNTs to the CFs/PA6 composites.

Grafting HCCP onto CFs improves their mechanical properties primarily by increasing interfacial adhesion between the fibers and the polymer matrix, such as PA6. The addition of HCCP promotes the formation of a cross-linked polyphosphazene layer on the fiber surface, increasing the surface energy and wettability of the fiber. This modification improves mechanical interlocking and provides a larger effective contact area between the CFs and the matrix, resulting in a stronger bond. As a result, the improved interfacial interactions reduce the possibility of debonding and failure under stress, significantly improving the overall mechanical performance of CF-reinforced composites.

The study discovered that adding hierarchical polyphosphazene nanotubes (PZSNTs) significantly increased the IFSS of CF reinforced polyamide 6 (CF/PA6) composites. The IFSS increased to 52.32 MPa, representing a 38.9% improvement over bare CF/PA6 composites and a 23.7% improvement over CFO/PA6 composites. This improvement is attributed to increased surface energy, improved wettability, and the formation of a loading transition layer, which aids in mechanical interlocking between the fibers and matrix. The initial decrease in IFSS caused by the removal of commercial sizing was effectively counteracted by the PZS coating, demonstrating the effectiveness of PZSNTs in improving composite interfacial adhesion.

**Figure 15:**
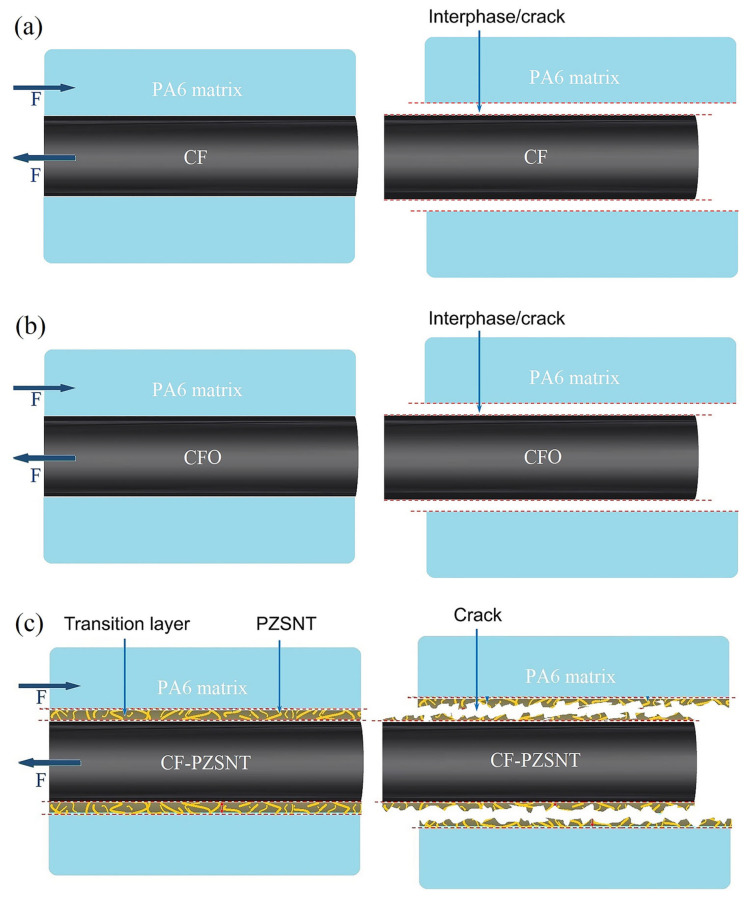
Proposed failure mechanism for CFs/PA6 polymer materials: (**a**) bare CF, (**b**) CFO, and (**c**) CF-PZSNTs [52]. Copyright 2023 Springer.

**Figure 16:**
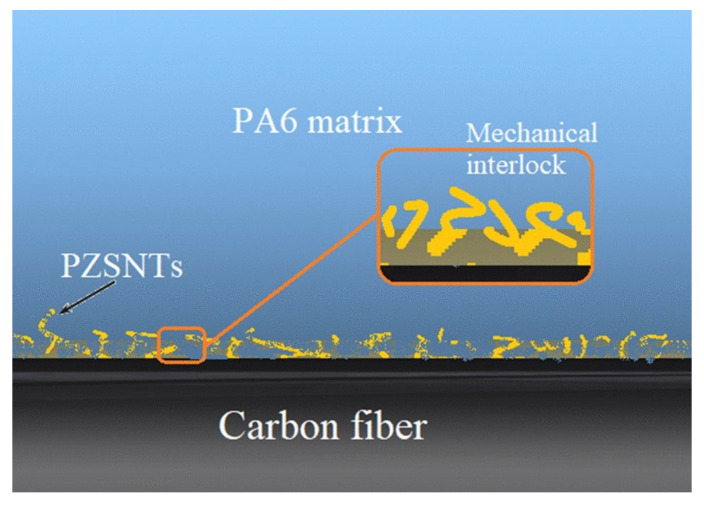
Schematic models for microscopic mechanical interlocking [52]. Copyright 2023 Springer.

Figure 15 depicts the interface region of CFs/PA6 materials, focusing on the mechanisms by which PZSNTs improve interfacial adhesion. The clean fracture surface of bare CFs/PA6 composites suggests a weak bond between the fibers and the PA6 matrix, resulting in interface failure (Figure 15a,b). In contrast, the debonding surface of CF-PZSNTs contains numerous fragments of the PZS layer and residues of PA6 resin (Figure 15c), indicating a shift toward a cohesive failure mode, as evidenced by the remaining PA6 on the fiber surface. The addition of PZSNTs to the CF surface forms a hierarchical composite interface, improving interfacial contact and increasing fracture area (Figure 16). Furthermore, the PZSNTs at the CF-PZSNTs/PA6 interface function as a physical force absorption layer, effectively transferring and dispersing stress, limiting crack propagation, and altering crack paths, thereby improving overall mechanical properties [53,56]. This demonstrates that CF-PZSNTs/PA6 composites have significantly stronger interface adhesion than unmodified CF/PA6 composites, which is due to higher surface energy, better wettability, and improved mechanical interaction between the PA6 matrix and the fibers.

Another study by the same group [53] studied into the effect of hybrid PPZ film treatment on CFs in CF/PA6 composites. This study investigated the interfacial properties of these polymer materials, as well as the possible benefits of the coating treatment. The main findings concern the interfacial properties of CF/PA6 materials after the hybrid polysphosphazene coating treatment. After applying the polysphosphazene coating, the IFSS of CF-PZSMS/PA6 composites increased by 42.75 percent. The enhanced interfacial adhesion was attributed to the combined effect of improved wettability, compatibility, stronger hydrogen bonds, and mechanical interlocking between the modified CFs and the PA6 resin matrix.

**Figure 17:**
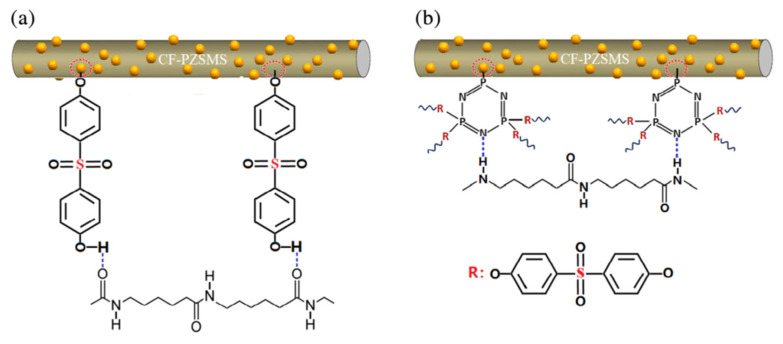
A chemical diagram of the hydrogen bonding between PA6 (**a**) and PZS (**b**) [53]. Copyright 2020 Wiley.

Improved interfacial properties resulted from the PZSMS coating’s increased mechanical interlocking and contact points between the modified CFs and the PA6 resin matrix. Firstly, the greatly enhanced surface energy can result in improved wettability between the PA6 matrix and CF-PZSMS, reducing defects at the composite interface. Secondly, the large number of N=P ring and -OH groups on the surface of PZS multiscale hybrid film modified CF allows for more and stronger H-bonding between the PA6 matrix. Indeed, due to the strong electron (e^−^) withdrawing characteristic of O=S=O groups, H in -OH groups has a higher electropositivity in BPS molecules. As a result, when compared to -OH groups on the CFO surface, H-bonds can be established more easily between -OH groups on the CF-PZSMS surface and C=O groups in the PA6 matrix (Figure 17a). On the other hand, the N atoms in the N=P ring can provide lone pair e^−^ [57], allowing H-bonds to form between the N=P ring and amide groups in the PA6 matrix (Figure 17b). As a result, in comparison to CFO, more and stronger H-bonds between modified CF and PA6 resin can be generated, hence improving interfacial adhesion significantly. Furthermore, PZSMS can adhere to the PA6 resin and act as an anchor, improving the mechanical interlocking effect and greatly increasing the interfacial shear strength of CFPZSMS/PA6 materials [58].

The presence of numerous hydrogen bonds between the modified CFs and the PA6 matrix helped to improve interfacial adhesion in the composites.

These findings imply that the hybrid polyphosphazene film treatment improves the interfacial properties of CF/PA6 materials, which could lead to the development of high-performance materials for a variety of applications.

**Figure 18:**
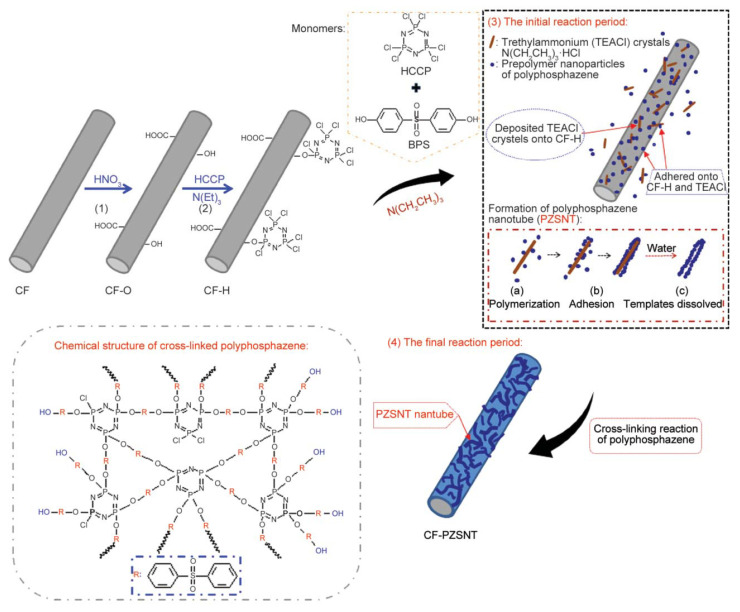
A schematic representation of the CF-PZSNT preparation process [54].

Zhang et al. [54] developed a straightforward method for producing needle-like PZSNT on CFs (CF-PZSNT) using in situ polycondensation under mild reaction conditions, resulting in a multiscale reinforcement. Figure 18 clearly illustrates the various synthesis techniques and processes.

The IFSS values for PA6 composites reinforced with various types of CFs show significant differences in interfacial properties. The virgin CF reinforced PA6 composites had an IFSS of 39.7 MPa. When CFs were oxidized (CF-O), the IFSS increased to 52.4 MPa, indicating a significant improvement due to the addition of oxygen-containing functional groups. The composites reinforced with polyphosphazene nanotubes grafted onto carbon fibers (CF-PZSNT) showed the most significant improvement, with an IFSS of 69.6 MPa. This represents a remarkable increase of approximately 75.3% over the virgin CF composites and 32.8% over the oxidized CF composites, demonstrating the effectiveness of the PZSNT’s multiscale reinforcement in improving interfacial bonding with the PA6 matrix.

**Figure 19:**
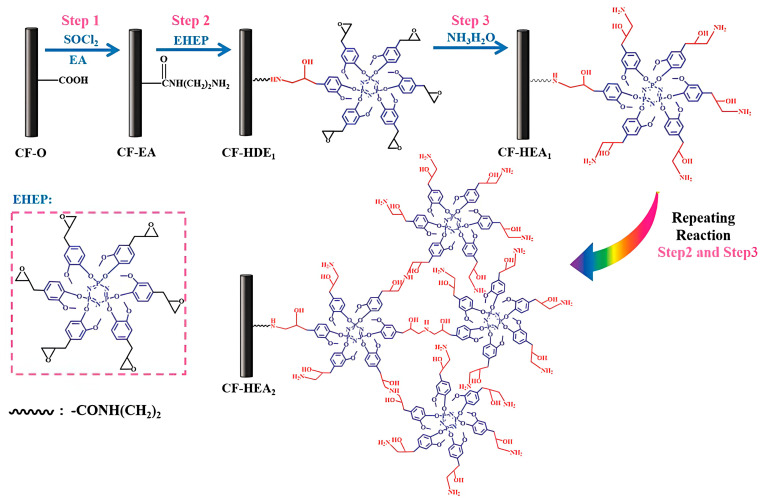
Schematic of branching polyphosphazene grafted onto CF [45].

He et al. [45] investigated a simple method for grafting bi-functional groups of terminated branching polyphosphazene (PPZ) onto carbon fibers via direct epoxy resin amine with an aqueous ammonia solution (NH_4_OH). The technique of the preparation is presented in Figure 19.

The technique for grafting bi-functional groups terminated branched polyphosphazene onto CFs is a multi-step process, as presented in Table 1.

The IFSS values for various CF/EP composites show a clear trend of increasing with different surface modifications. The IFSS for untreated carbon fiber/epoxy (CF-O/EP) composites was measured at 43.6 MPa. In contrast, composites containing amino-functionalized carbon fibers (CF-EA/EP) have an IFSS of 65.3 MPa. The introduction of branched polyphosphazenes results in further enhancements, with CF-HEA1/EP composites achieving an IFSS of 76.8 MPa and CF-HEA2/EP composites exhibiting the highest IFSS value of 89.6 MPa. This progression demonstrates the utility of surface modifications in improving the interfacial properties of CF/EP composites.

**Figure 20:**
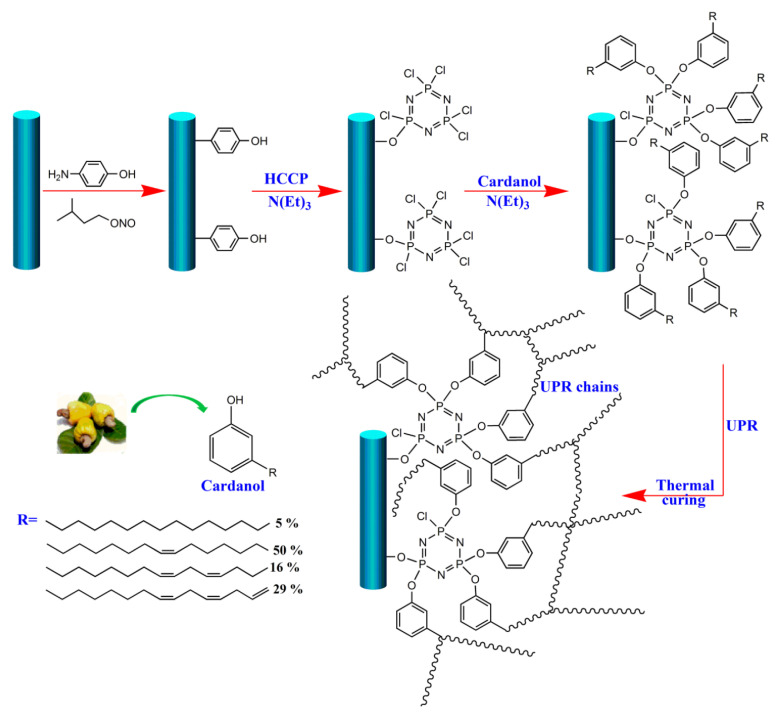
A schematic illustration of the CF-cardanol production technique as well as the interfacial reaction between the matrix and modified CFs [46].

Zheng et al. [46] successfully introduced cardanol onto carbon fiber surfaces through a series of well-defined steps. Figure 20 depicts a schematic depiction of the entire functionalized approach for CF-cardanol. The synthetic technique for modifying CF surfaces with renewable cardanol is presented in Table 1.

The composites’ interfacial property testing revealed significant improvements in ILSS as a result of cardanol modification of carbon fibers. The ILSS value for untreated CF composites was 48.2 MPa. Following treatment with hydroxyl groups (CF-OH), the ILSS increased to 57.68 MPa, representing a 19.67% increase. The CF-cardanol composites showed the most significant improvement, with an ILSS of 68.13 MPa, representing a 41.35% increase over untreated composites and an 18.11% increase over CF-OH composites. This improvement is due to the modified fibers’ hierarchical structure and increased surface roughness, which enabled better mechanical interlocking and chemical bonding with the matrix resin during the curing process.

**Figure 21:**
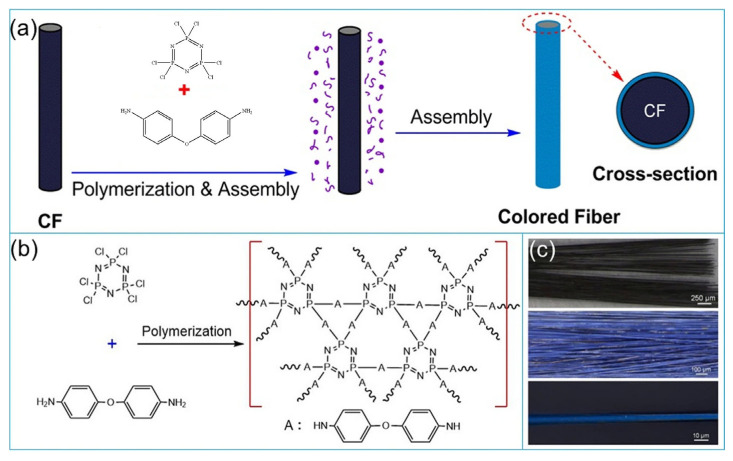
Schematic for CF structural coloration (**a**), schematic for preparing poly(cyclotriphosphazene-co-4,4′-oxydianiline) layer (**b**), and original black and colored CF (**c**) [55]. Copyright 2024 Elsevier.

Xu et al. [55] successfully colored CF using thin-film interference by in situ building a layer of poly(cyclotriphosphazene-co-4,4′-oxydianiline) layer on the CF. The technique of the preparation is presented in Figure 21.

According to the study’s tensile strength results, the desized CF had a tensile strength of 4.65 GPa, while the oxidized fiber (CFO) had a lower strength of 4.28 GPa due to oxidation induced surface defects. In contrast, the colored CF had a tensile strength of 5.01 GPa, which was 7.7% higher than the desized CF and 17.1% higher than the oxidized fiber. Furthermore, the colored CF’s breaking elongation was measured at 2.33%, which exceeded both the CFO (1.87%) and the original CF (1.98%), demonstrating the improved mechanical properties obtained through the structural coloration process.

**Figure 22:**
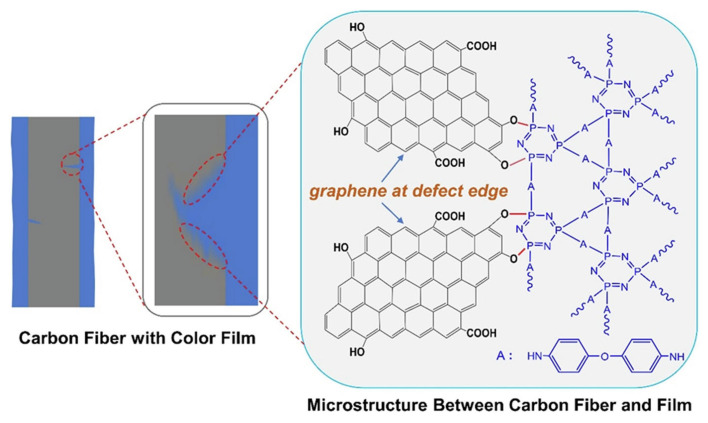
Schematic of the microstructure formed by CF and polyphosphazene sheet [55]. Copyright 2024 Elsevier.

**Figure 23:**
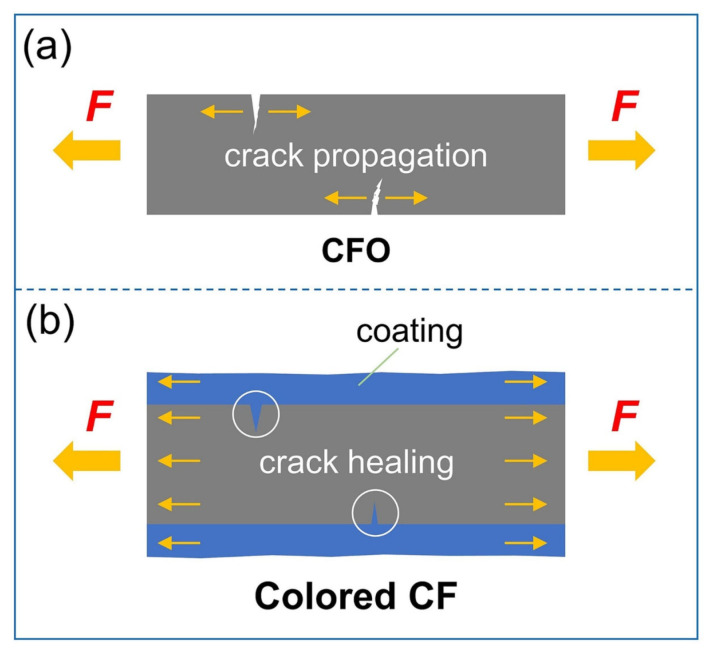
Schematic depicting the mechanical characteristics of CFO (**a**) and colored CF (**b**) during tensile testing [55]. Copyright 2024 Elsevier.

Figure 22 depicts the interaction and microstructure of CFs and polyphosphazene films, emphasizing the healing process and mechanism involved. Initially, during in situ polymerization, active oligomers or nano nuclei particles with P-Cl or –NH_2_ groups are formed, which adhere to fiber surfaces and fill surface imperfections via physisorption and/or chemisorption. Physisorption can occur due to changes in surface energy, whereas chemisorption is caused by SN between P-Cl and phenolic hydroxyl groups (-OH) on fiber surfaces. As polymerization proceeds, these active oligomers aggregate on the CF surfaces and within surface flaws, effectively repairing defects and forming a crosslinked polyphosphazene sheath around the fibers. This crosslinking is initiated by nucleophilic substitution between the active oligomers’ –NH_2_ groups and P-Cl, allowing the crosslinked polyphosphazene layer to act as a protective covering that absorbs axial stress while inhibiting fracture initiation and propagation, as seen in Figure 23. Colored CF-reinforced EP materials exhibit a significant increase in IFSS, which rises from 50.1 to 74.5 MPa (a 48.7% increase) compared to desized fiber (CF) and from 56.6 to 74.5 MPa (a 31.6% increase) compared to CFO. The fracture morphologies of the colored CF-reinforced EP composite show a rough surface with significant residual resin tightly adhering to the debonded surface of the colored fiber, indicating strong interfacial adhesion. This improvement in IFSS is thought to be caused by the presence of amines on the coloration layer’s surface.

**Figure 24:**
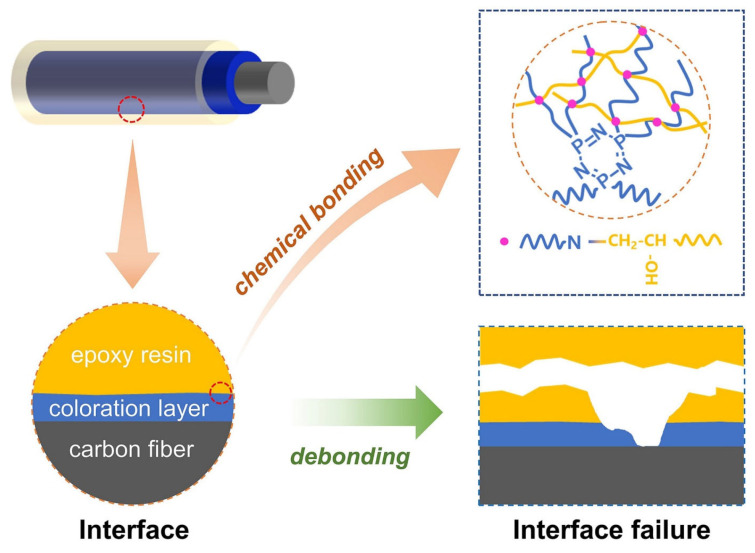
Schematic of the interface microstructure of a colored CF/EP material and its failure mode [55]. Copyright 2024 Elsevier.

During the curing phase, amines on colored CF surfaces created strong chemical bonds with the epoxy resin matrix [59] (Figure 24). These strong chemical bonds greatly improve the surface adhesion of colored CF/EP composites. The composite contact is strong enough to transfer shear loads from the matrix to the fiber. The initiation and propagation of microcracks under shear pressure occurred mostly in the inner resin matrix and interface region, culminating in fracture failure of both the resin matrix and coloring film.

These findings show that the colored CF-reinforced EP composite has superior interface properties, which leads to better mechanical properties and possible applications in advanced composite materials.

### 2.2. Silicon Grafting onto CF Composites

Silica nanoparticles (SiO_2_) are regarded as promising polymer composite reinforcements due to their unique spherical structure and excellent physicochemical properties, particularly the abundance of surface silanol groups, which improve fiber surface polarity and facilitate chemical bonding with the matrix. According to research, incorporating SiO_2_ into CF/phenolic nanocomposites (NCs) improves mechanical and thermal stability by increasing dispersion and interfacial adhesion. Furthermore, the formation of silicon carbide at high temperatures increases ablation resistance. Studies using SiO_2_ and graphene oxide (GO) in EPs have shown that synergistic effects improve impact strength, tensile properties, and thermal performance. The sol-gel process for producing SiO_2_ NPs is both efficient and cost-effective, making it suitable for large-scale applications. However, the chemical grafting of SiO_2_ onto CF surfaces to form SiO_2_/CF reinforcements has received little attention, despite its potential to significantly improve interfacial strength via covalent bonding and mechanical interlocking [60].

**Figure 25:**
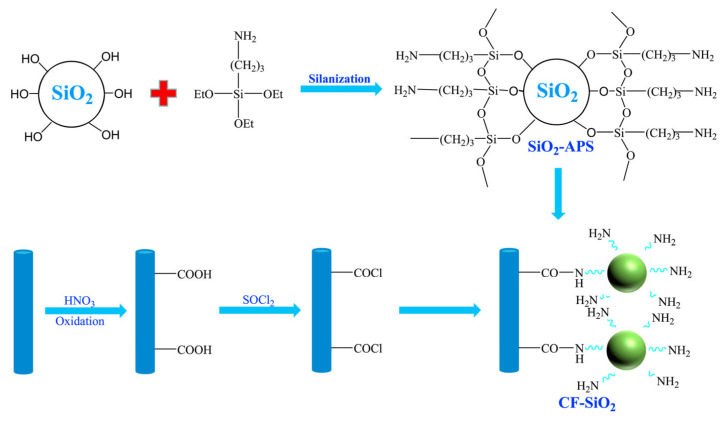
The synthesis procedures for SiO_2_-APS and CF-g-SiO_2_ [60]. Copyright 2024 Elsevier.

Wu et al. [60] developed a simple and effective chemical strategy to improve the interfacial strength of silicone resin composites by chemically grafting silica NPs onto CFs. The formation of SiO_2_ grafted onto CF was accomplished in two steps, as shown in Figure 25.

This study described a chemical grafting method for attaching SiO_2_ to CFs. Specifically, APS functionalized silica NPs (SiO_2_-APS) were synthesized and covalently linked to the CF surfaces. This process involved a reaction between the amino groups of SiO_2_-APS and the chloride groups on the CF surface. FTIR and XPS analyses confirmed the successful grafting, revealing changes in the chemical composition and functional groups on the fiber surface as a result of the grafting process. This technique focuses on enhancing the interface between carbon fibers (CFs) and the polymer matrix in composites by increasing surface polarity and establishing strong mechanical interlocking.

The incorporation of SiO_2_ nanoparticles (NPs) into CFs leads to a significant improvement in the ILSS of the resulting composites, with ILSS values rising by 53.27% compared to untreated CF composites. This enhancement is attributed to the formation of covalent bonds between SiO_2_-aminopropyltriethoxysilane (APS) and the CF surface, which improves interfacial adhesion to the polymer matrix. Additionally, the modification enhances mechanical interlocking and wettability, resulting in better compatibility with the matrix. The grafting process increases surface roughness, contributing to a stronger interface, which results in a notable increase in ILSS from 41.08 MPa for untreated fibers to 63.00 MPa for modified fibers.

**Figure 26:**
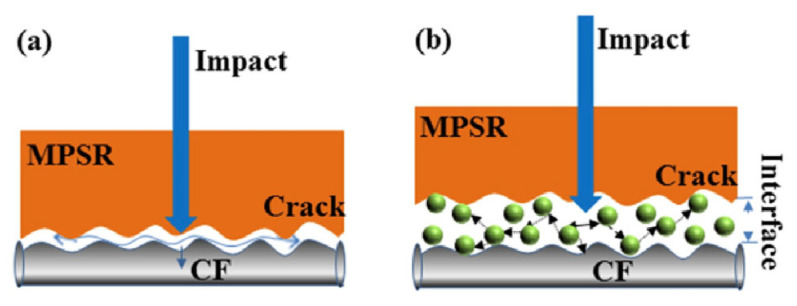
Schematic of impact testing of composites reinforced with (**a**) untreated CF and (**b**) CF-g-SiO_2_ [60]. Copyright 2024 Elsevier.

The modification of CFs had a significant impact on the composites’ impact properties, as evidenced by the test results. Composites reinforced with untreated CFs had an impact strength of 58.46 kJ/m^2^, but increased to 68.06 kJ/m^2^ when modified with APS (CF-APS). Modifying the composites with SiO_2_-APS increased their impact toughness to 74.31 kJ/m^2^ for CF-ad-SiO_2_ and 78.89 kJ/m^2^ for CF-g-SiO_2_. This increase in impact strength, attributed to the presence of the SiO_2_-APS interface, suggests that the modified fibers provide a shielding effect that prevents crack propagation, thus improving the composites’ overall toughness and durability. Figure 26a,b show a schematic illustration of the composites interface.

## 3. Reactive Flame Retardant Agents

The high-performance flexible NCs developed in this study have numerous applications, particularly in electronics and electrical equipment, where they can serve as effective electromagnetic interference (EMI) shielding materials. Their superior fire safety and mechanical properties make them ideal for use in advanced communication systems, automotive electronics, aerospace engineering, and medical devices. Furthermore, the lightweight and corrosion-resistant properties of these nanocomposites make them promising alternatives to traditional metal-based shielding materials, allowing for easier integration into next-generation technologies such as 5G facilities.

**Figure 27:**
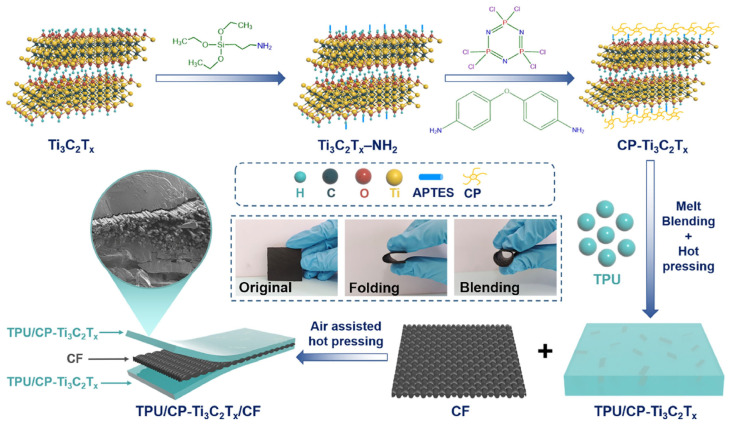
Schematic diagram for fabricating TPU/CP-Ti_3_C_2_T_x_/CF NCs [61]. Copyright 2023 Elsevier.

Liu et al. [61] successfully fabricated a novel hierarchical thermoplastic polyurethane (TPU)/cyclophosphazene-functionalized Ti_3_C_2_T_x_/CF fabric (TPU/CP-Ti_3_C_2_T_x_/CF) nanocomposite. Figure 27 shows the process of preparing TPU/CP-Ti_3_C_2_T_x_/CF NCs.

**Figure 28:**
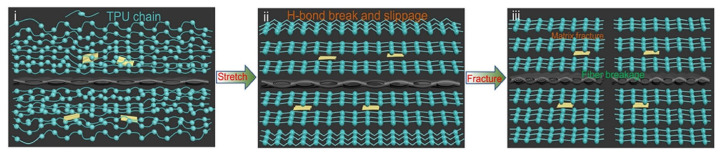
Schematic representations of the mechanical failure process models of TPU/CP-Ti_3_C_2_T_x_/CF: (**i**) Before Stretching (ε = 0), (**ii**) During Stretching (0 < ε < ε_b_), and (**iii**) At Break Point (ε = ε_b_) [61]. Copyright 2023 Elsevier.

The incorporation of 1.0 wt.% CP-Ti_3_C_2_T_x_ into TPU/CF nanocomposites resulted in significant enhancements in mechanical, thermal, and flame retardant properties. The tensile strength increased from 39.3 MPa to 49.1 MPa, marking a 24.9% improvement, although a higher loading of 4.0 wt.% led to a slight decrease in tensile strength to 40.3 MPa. Additionally, the elongation at break improved from 6.7% to 8.7%, representing a 30.0% increase, while tensile toughness doubled from 1.1 MJ/m^3^ to 2.2 MJ/m^3^. The mechanical failure process of the TPU/CP-Ti_3_C_2_T_x_/CF nanocomposites is characterized by a strong 3D structure formed through hydrogen bonding, which facilitates elastic deformation and stress whitening under tensile stress (Figure 28). As the material is loaded, microcracks develop at stress concentration points, which propagate and merge into macrocracks, ultimately leading to failure.

**Figure 29:**
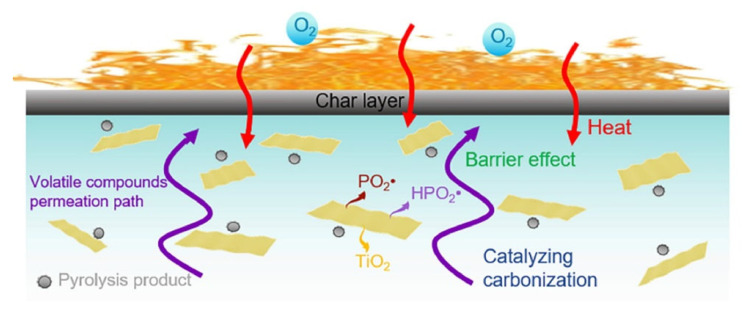
Flame retardant mechanism of TPU/CP-Ti_3_C_2_Tx NCs [61]. Copyright 2023 Elsevier.

The TPU/CP-Ti_3_C_2_T_x_ nanocomposites exhibit excellent thermal stability, with CP-Ti_3_C_2_T_x_ showing only 70.35 wt.% weight loss at 800 °C, outperforming neat TPU. The flame retardancy of the composites is significantly enhanced, as the formulation with 4.0 wt.% CP-Ti_3_C_2_T_x_ reduces the PHRR and THR by 64.4% and 31.8%, respectively, compared to pure TPU (Table 3). The proposed flame retardant mechanism involves a multifaceted approach to improve thermal stability and reduce flammability (Figure 29). CP-Ti_3_C_2_T_x_ serves as a physical barrier that minimizes heat transfer and prevents ignition of the TPU matrix. Its catalytic carbonization ability promotes the formation of a stable char layer during combustion, which acts as an insulator, reducing heat and mass transfer while releasing phosphorus-containing species that disrupt combustion by generating free radicals. This action effectively terminates chain reactions in the combustion zone. Additionally, the incorporation of CFs enhances the structural integrity of the char, further improving the composite’s flame retardancy. The synergistic effects of physical barrier formation, catalytic action, and radical scavenging underscore the effectiveness of CP-Ti_3_C_2_T_x_ as a flame retardant in TPU-based materials, making them suitable for applications that demand high fire safety standards.

**Figure 30:**
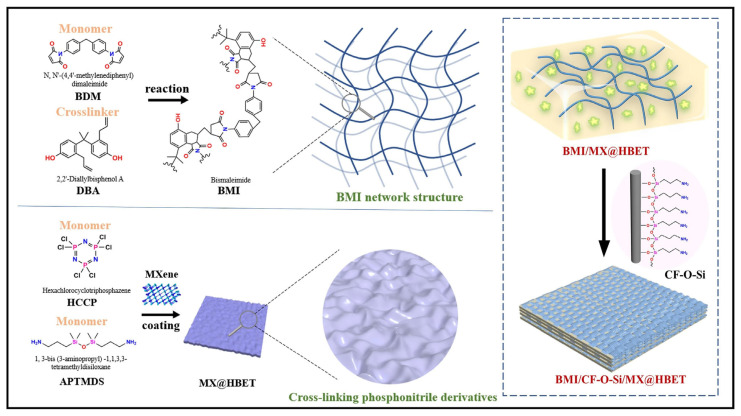
A diagram depicting for the synthesis of BMI/CF-O-Si/MX@HBET [62]. Copyright 2024 Elsevier.

Ye et al. [62] used APTMDS and HCCP to form a crosslinked network on the surface of MXene to produce a new flame retardant (MX@HBET). Figure 30 depicts the novel synthesis and processing approach. BMI/CF-O-Si/MX@HBET is created in a series of stages that incorporate the various components, resulting in a composite material with improved fire safety and mechanical properties.

The study demonstrates that using MXene-based hybrid materials and silane-modified CFs significantly improves BMI composites’ fire safety and mechanical strength. Adding 1 wt.% MX@HBET to BMI/CF composites reduced THR by 10.73% and TSP by 33.3% in combustion tests. When exposed to a 1500 °C butane flame, the laminates maintained a backplane temperature of around 430 °C for 20 min. The composites’ impact strength increased to 82.10 kJ/m^2^ and tensile strength reached 466.6 MPa, compared to 63.52 kJ/m^2^ and 350.1 MPa for pure BMI/CF composites, indicating a significant improvement in toughness and rigidity due to the synergistic effects of the modified materials (Table 3). These findings show that MXene-based hybrid materials have tremendous promise for improving both fire safety and mechanical strength in composite materials, especially in applications that require high-performance materials with increased fire resistance properties.

**Figure 31:**
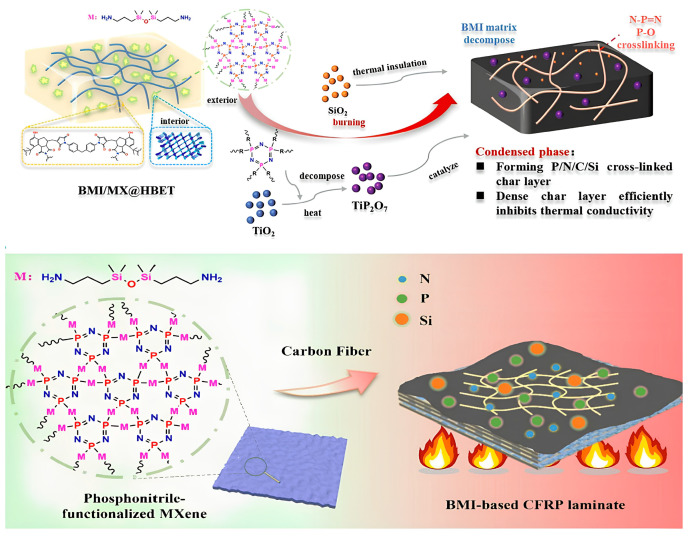
Flame retardant mechanism of BMI/MX@HBET [62]. Copyright 2024 Elsevier.

The fire-retardant mechanism of BMI and its composites, particularly those containing MX@HBET, includes a number of critical reactions and interactions that work together to improve fire resistance. Based on the results, a possible fire protection mechanism is shown in Figure 31. Char formation is a key mechanism in which the BMI resin generates a protective char coating during combustion, and MX@HBET significantly improves on this process. Additionally, the incorporation of organic silicon components in the composites leads to the formation of SiO_2_ during combustion, which enhances the char layer and serves as a barrier against heat and flame propagation. The catalytic effects of phosphonitrile and titanium-containing compounds in MX@HBET promote the formation of the char layer, while simultaneously reducing flammability and enhancing fire resistance. Moreover, the use of MX@HBET results in lower PHRR and THR, effectively managing fire intensity and limiting flame spread. The barrier properties of the char layer also contribute to a reduction in TSP. Ultimately, MX@HBET enhances the thermal stability of BMI composites, allowing them to endure high temperatures and fire exposure, making them well-suited for applications that demand high fire resistance.

Glaskova-Kuzmina et al. [63] explored innovative modifications to EP composites that markedly enhance both fire resistance and mechanical performance. Their study on EP composites modified with HCCP yielded valuable insights into improving fire resistance. Notably, the addition of HCCP resulted in a significant reduction in after-flame times during vertical burn tests, with an optimal content of 7.2 wt.%, which significantly improved flame-retardant properties when compared to unmodified EP samples. The vertical burn tests revealed that the HCCP-modified composites had significantly shorter after-flame times than the unmodified samples; whereas the unmodified EP completely burned during the after-flame period, the modified samples exhibited a flame that extinguished after this period, leaving a residual length of 6–9 cm. Specifically, composites containing 7.2 wt.% HCCP showed a reduction in after-flame times of approximately 38% for glass fiber-reinforced composites (FRC), 27% for carbon FRC, and an impressive 57% for basalt FRC compared to their neat counterparts. Furthermore, the study indicated that increasing the HCCP content to 9.5 wt.% further enhanced fire resistance, although this higher concentration could lead to a decline in mechanical properties.

## 4. Combined Approaches for Sustainability, Fire Safety, and Recyclability

**Figure 32:**
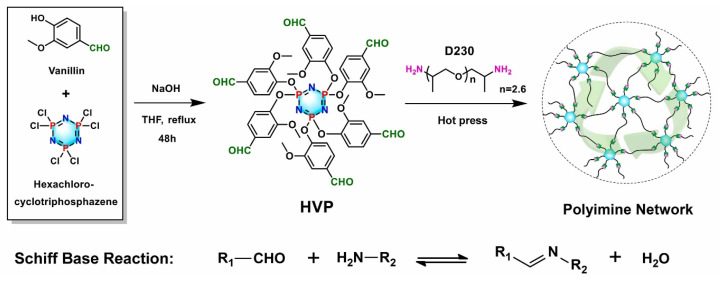
The Schiff base reaction is used to synthesize flame-resistant monomers HVP and HVP/D230 polyimine [47]. Copyright 2021 Elsevier.

The materials science community has been increasingly focused on recent advancements in CFRP composites that are sustainable, flame-retardant, and recyclable. Researchers are striving to enhance the environmental sustainability and safety of these high-performance materials while maintaining their exceptional mechanical properties. One promising approach is to modify CF surfaces and improve interfacial properties using renewable resources like cardanol extracted from cashew nut shell liquid. This bio-based modification not only improves the composite’s overall performance but also promotes sustainability. Novel additives and surface treatments are being used to address flame retardancy, while thermoplastic matrices and reversible cross-linking systems are being developed to improve recyclability. These advancements aim to address CFRP composites’ traditional limitations, such as poor flame resistance and end-of-life disposal issues, while also lowering their environmental impact and expanding their potential applications in a variety of industries. Liu et al. [47] investigated how to achieve both flame retardancy and full recyclability of CFRPs using newly designed flame-retardant and readily degradable polyimine thermosets as binders to construct CFRPs made from a synthesized vanillin-terminated phosphazene monomer (HVP) and commercial polyetherimide D230 without a catalyst (Figure 32 and Table 1).

**Figure 33:**
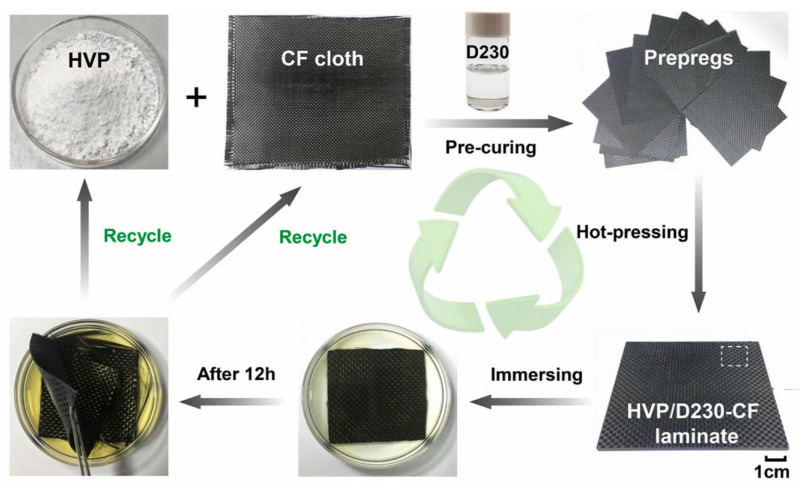
Schematic diagram for preparing and recycling HVP/D230-CF [47]. Copyright 2021 Elsevier.

HVP/D230 serves as a binder in the HVP/D230-CF composite due to its recyclability and high flame retardant properties (Figure 33). The preparation process involves dissolving HVP in dichloromethane, adding D230 in a specific ratio, and curing the mixture. The presence of degradable imine bonds in the polyimine thermosets enhances the composite’s recyclability, enabling non-destructive closed-loop recycling under mild conditions while preserving performance. Additionally, the dynamic crosslinking of imine bonds allows for exceptional reparability; the composite can be repaired through hot pressing at high temperatures, achieving over 90% restoration of flexural strength and 75% of modulus. The HVP/D230-CF material also exhibits excellent creep resistance, crucial for maintaining dimensional stability and mechanical properties over time, attributed to its increased crosslinking density. The mild and effective closed-loop recycling technique regenerates composite laminates without performance loss, as the regenerated composite retains flexural properties comparable to the original, demonstrating the efficiency of the recycling process.

The flame retardancy of the HVP/D230-CF material is assessed through various tests, demonstrating its superior fire-resistant properties. It has a significantly higher LOI value of 34.2% compared to the standard E51/D230-CF, which has an LOI of 21.1%. Additionally, HVP/D230-CF achieves a UL-94 rating of V-0, indicating it meets high flame retardancy standards by being able to withstand combustion and self-extinguish (Table 3). Cone calorimeter tests further reveal that HVP/D230-CF exhibits lower PHRR, THR, and PSPR values than E51/D230-CF, signifying improved flame retardancy and reduced flammability. During combustion, the composite generates a charred residue that serves as a protective barrier, slowing flame spread and minimizing heat release, thereby enhancing its flame retardant properties. Overall, HVP/D230-CF outperforms conventional commercial materials in flame retardancy, as evidenced by its V-0 rating in the UL-94 test and superior performance in cone calorimeter tests, effectively preventing fire propagation.

**Figure 34:**
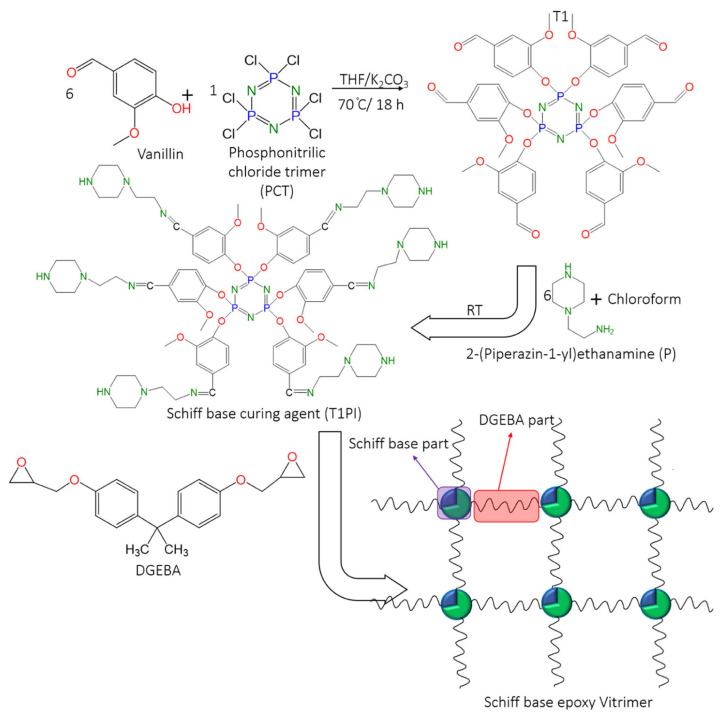
T1PI and T1PI-DGEBA curing synthesis route [48]. Copyright 2024 ACS.

Zamani et al. [48] explored the creation of high-performance carbon fiber-reinforced epoxy vitrimer composites that are flame-retardant and recyclable. They developed a novel curing agent, T1PI, derived from vanillin, which enhances the properties of these composites. The synthesis steps of T1PI are presented in Figure 34 and Table 1.

**Figure 35:**
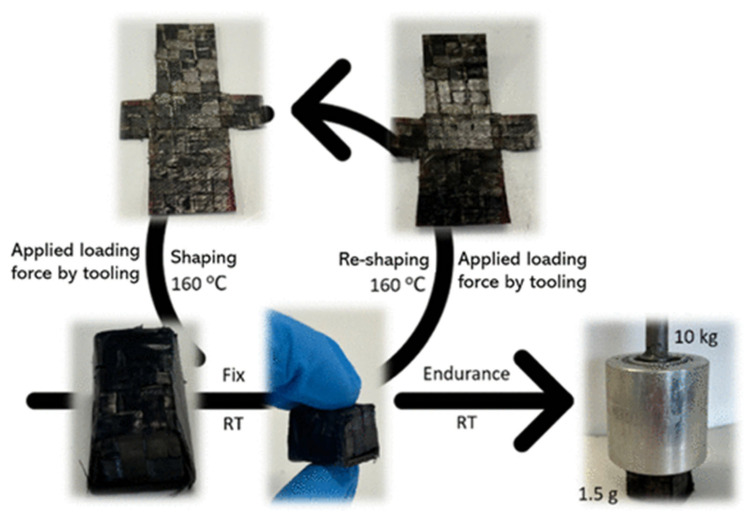
Illustration of the loading force-induced thermoformability of Schiff base CFRP [48]. Copyright 2024 ACS.

The study highlights the exceptional thermoformability of the Schiff base CFRP composite, T1PI/DGEBA, which can be reshaped with heat and applied pressure (Figure 35). A square sample was transformed into a four-armed petal shape and then molded into a cube at 160 °C, demonstrating its ability to retain the new shape at RT, showcasing its mechanical strength and structural integrity. The dynamic Schiff base bonds in the epoxy cross-linking network facilitate rapid chain reconfiguration when heated, allowing significant shape changes while maintaining performance.

**Figure 36:**
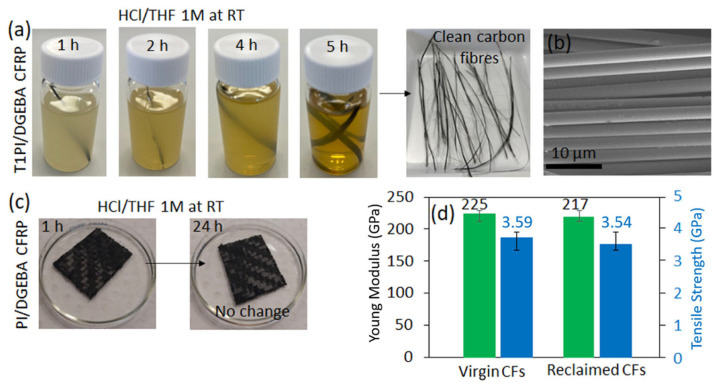
(**a**) T1PI/DGEBA CFRP degradation in 1 M HCl/THF (2:8, *v*/*v*) solution; (**b**) SEM image of reclaimed CF surface; (**c**) degradability of PI/DGEBA CFRP in 1 M HCl/THF (2:8, *v*/*v*) solution over time; and (**d**) mechanical properties of virgin and reclaimed CFs [48]. Copyright 2024 ACS.

Additionally, the study examined the degradability of T1PI/DGEBA CFRP in a 1 M HCl/THF solution, finding that it can completely dissolve in 5 h due to the uniform distribution of Schiff base bonds, unlike the PI/DGEBA CFRP, which remained intact after 24 h (Figure 36). SEM images of reclaimed CFs indicated that the recycling process preserves their structural integrity, with mechanical properties of reclaimed fibers comparable to virgin fibers, suggesting effective recycling without significant performance loss.

The successful synthesis of T1PI, a multifunctional secondary amine curing agent from vanillin, incorporates dynamic Schiff base bonds into the epoxy network. This curing agent effectively cures DGEBA, resulting in a high-performance CFRP composite (T1PI/DGEBA) with impressive mechanical properties, including a tensile strength of 490 MPa and a T_g_ of 139 °C. The composite exhibits excellent thermal stability and fire retardancy, achieving a UL-94 V0 rating and a LOI of 31.5%, indicating superior combustion resistance compared to traditional epoxy systems. Furthermore, T1PI/DGEBA cures at lower temperatures, extending the shelf life of prepregs, and its high nitrogen and phosphorus content enhances chemical recyclability, allowing for the recovery of CFs under mild conditions. These findings emphasize T1PI’s potential as an efficient curing agent for durable, high-performance CFRP composites suitable for various engineering applications.

## 5. Applications in Composite Materials

Carbon fiber-based polymer composites are extensively utilized in high-technology sectors, particularly in aerospace, where their lightweight and high-strength properties make them ideal for constructing critical aircraft components, including wings and fuselage structures. In the automotive industry, these composites are favored for manufacturing high-performance and durable parts, such as body panels and structural elements. Their excellent fatigue and corrosion resistance also enable applications in sports equipment, wind turbine blades, marine vessels, and construction, where they reinforce structures. The increasing demand for lightweight, high-strength materials across these diverse fields highlights the versatility and significance of carbon fiber composites in modern engineering solutions [10]. Similarly, the hierarchical TPU/CP-Ti_3_C_2_T_x_/CF nanocomposites developed by Miao Liu et al. [47] demonstrate substantial potential in electronics and electrical devices, offering high fire safety, exceptional mechanical strength, and effective electromagnetic interference (EMI) shielding. These attributes render them ideal for use in advanced electronic devices, 5G infrastructure, and other technologies where safety and performance are crucial. Their lightweight and corrosion-resistant nature positions them as viable alternatives to conventional metal-based EMI shielding materials, expanding their use in aerospace, automotive electronics, and medical applications. Additionally, the MXene-based hybrid nanocomposites developed by Ye et al. [62] are especially significant in the aerospace sector, where improved fire resistance and mechanical strength are essential. These nanocomposites are well-suited for structural applications in aircraft and spacecraft, capable of enduring extreme temperatures and mechanical stresses while reducing fire risks. Their outstanding thermal insulation properties also make them suitable for protective coatings and components in high-temperature settings, with potential applications in the automotive, construction, and other industries that demand lightweight, durable, and fire-resistant materials for enhanced safety and performance.

## 6. Conclusions

Recent advances in flame retardant technologies for EPs have significantly improved the fire safety and performance of composite materials, especially in high-demand applications. Chemical grafting of flame-retardant compounds onto CFs is a significant innovation that integrates fire-resistant functionalities directly at the fiber–matrix interface. This method not only increases thermal stability and reduces smoke toxicity, but it also improves interfacial adhesion and overall composite performance. The introduction of reactive flame-retardant agents, such as phosphorus-based compounds, silicone, and nitrogen-rich polymers, has improved EP matrices by providing long-lasting, intrinsic flame-retardant properties without compromising mechanical integrity. These technologies have been successfully applied in critical industries such as aerospace, automotives, and renewable energy, addressing stringent safety and regulatory requirements. For example, composite pressure vessels and structural components have demonstrated significant improvements in fire resistance and longevity. Furthermore, the use of bio-based flame retardants aligns with sustainability objectives, reducing reliance on toxic additives and promoting environmentally friendly solutions. This combination of chemical grafting techniques, reactive flame retardants, and bio-based approaches is pushing the boundaries of high-performance, sustainable, and safer composite materials, meeting the growing demand for advanced materials that balance safety, performance, and environmental concerns across a wide range of industries.

## 7. Challenges and Future Prospects

HCCP grafting onto CF composites presents several challenges but also the challenges of HCCP grafting onto CF composites include achieving uniform and controlled grafting, optimizing reaction conditions, and scaling up for industrial use. However, it has significant future potential for improving fiber–matrix interfaces, developing eco-friendly techniques, and producing multifunctional composites for the aerospace, automotive, and renewable energy sectors. Challenges for bio-based flame retardants include mechanical property compromises, economic feasibility, and a lack of comprehensive real-world performance studies. The future focuses on interdisciplinary research, advanced testing methodologies, and application customization. CFRP recycling faces challenges due to complex material composition, high costs, and lower regenerated fiber quality. However, advances in materials science, innovative recycling technologies, and rising demand for sustainable solutions make the future look promising. The development of sustainable CFRP composites faces challenges such as optimizing curing processes, ensuring long-term durability, and improving recyclability. Future research should concentrate on effective carbon fiber reclamation methods, the economic feasibility of large-scale production, and integration into existing manufacturing processes.

## Figures and Tables

**Table 1:** A summary of the synthesis steps for HCCP-CF composites.

Samples	Steps	Ref.
CF-ACP	The process for modifying CFs involves multiple steps to enhance their properties. Initially, the sizing is removed through acetone refluxing, followed by fiber oxidation using nitric acid. The CF-O then undergo two grafting steps with HCCP, with the second step also incorporating DDE. Between each major step, the fibers are washed and dried. The final product, CF-ACP, is ultrasonically washed with water and DMF, then vacuum-dried.	[42]
CF-g-EG	The process for creating CF-g-EG composite involves a series of steps that combine CF and graphene-based materials. It begins with the production of GO, which is then modified using glycidol and HCCP. Separately, CFs are modified to create CF-ACP. The final step involves combining the CF-ACP with exfoliated graphene and additional reagents, resulting in the CF-g-EG composite.	[43]
CF/PPBES	The CF/PPBES composite creation process consists of two main stages: (1) Surface modification of CFs to produce CF-PPM, involving sizing removal, hydroxyl group introduction, HCCP reaction, and MA grafting; (2) Composite preparation, which includes dissolving PPBES resin, soaking CF tows, preparing monolayers, hot-pressing, and controlling CF volume fraction.	[44]
CF-HEA1/CF-HEA2	The preparation of CF-HEA1/CF-HEA2 involves four main steps: (1) oxidizing CFs to create CF-O, (2) grafting EDA onto CF-O to produce CF-EA, (3) grafting EHEP onto CF-EA to form CF-HDE1, and (4) performing epoxy amination on CF-HDE1 to yield the final products.	[45]
CF-cardanol	The process of modifying CFs with cardanol involves several steps. Initially, hydroxyl groups are introduced to the CFs using isopentyl nitrite and 4-aminophenol. The fibers are then washed and reacted with HCCP, followed by grafting with cardanol. After washing, the modified fibers are used to prepare a prepreg with unidirectional fiber orientation. The composite is then molded using compression molding, cured through a multi-stage thermal treatment under pressure, and finally cooled naturally while maintaining pressure.	[46]
HVP	HVP is synthesized through a NS reaction involving vanillin and anhydrous potassium carbonate in dry THF, followed by the dropwise addition of HCCP and heating at 70 °C for 48 h. After filtering the solid, the product is obtained by evaporating the solvent and recrystallizing with ethanol. The HVP/D230 polyimine is then created by dissolving HVP in dichloromethane and adding D230 in a 1:3 molar ratio, followed by solvent evaporation and hot pressing at 120 °C and 18 MPa.	[47]
T1PI	The synthesis of T1PI involves two steps: First, T1 is created by reacting vanillin with phosphonitrilic chloride trimer (PCT) in THF with K_2_CO_3_, forming a cyclophosphazene compound. In the second step, T1PI is produced through a Schiff base reaction between T1 and 2-(piperazin-1-yl)ethanamine, resulting in a multifunctional secondary amine curing agent.	[48]

**Table 2:** A summary of the IFSS and mechanical parameters of several reported HCCP-CF composites.

Samples	IFSS (MPa)	Flexural Strength (MPa)	Flexural Modulus (GPa)	Tensile Strength (GPa)	Ref.
CF-PP	10.2	-	-	-	[42]
CF-O/PP	9.8	-	-	-	
CF-ACP/PP	11.1	-	-	-	
CF-M-PP	10.0	-	-	-	
CF-O/M-PP	13.0	-	-	-	
CF-ACP/M-PP	32.3	-	-	-	
CF/EP	47.3	-	-	-	[43]
CFO/EP	57.2	-	-	-	
CF-ACP/EP	81.8	-	-	-	
CF-g-EG/EP	97.7	-	-	-	
CF/PPBES	77.8 (ILSS)	1598	119.2	-	[44]
CF-OH/PPBES	89.2 (ILSS)	1824	137.6	-	
CF-PPM/PPBES	95.6 (ILSS)	2067	156.1	-	
CF	47.5	-	-	4.80	[39]
CFO	56.6	-	-	4.69	
CFO-HCCP-ODA	81.3	-	-	4.74	
CF	47.5	-	-	4.80	[49]
CFO	56.6	-	-	4.69	
CF-ACP	81.0	-	-	5.31	
CF	68.91	-	-	4.55	[50]
CFO	70.18	-	-	4.17	
CF-PZSMS	84.75	-	-	4.82	
Received CF	73.51	-	-	-	[51]
Desized CF	68.91	-	-	4.55	
CFO	70.18	-	-	4.17	
CF-PZSNT	87.10	-	-	4.85	
Received CF	39.18	-	-	-	[52]
Bare CF	37.68	-	-	-	
CFO	42.28	-	-	-	
CF-PZSNTs	52.32	-	-	-	
CF	37.68	-	-	-	[53]
CFO	42.28	-	-	-	
CF-PZSMS-L	48.86	-	-	-	
CF-PZSMS-M	53.79	-	-	-	
CF-PZSMS-H	49.72	-	-	-	
CF	39.7	-	-	-	[54]
CF-O	52.4	-	-	-	
CF-PZSNT	69.6	-	-	-	
CF-O/EP	43.6	-	-	-	[45]
CF-EA/EP	65.3	-	-	-	
CF-HEA_1_/EP	76.8	-	-	-	
CF-HEA_2_/EP	89.6	-	-	-	
CF	50.1	-	-	4.65 ± 0.90	[55]
CFO	56.6	-	-	4.28 ± 1.17	
Colored CF	78.9	-	-	5.01 ± 1.14	

**Table 3:** Comparison of the mechanical, thermal, and flame retardancy properties of HCCP-CF composites.

Matrix	*T*_max_ (°C)	LOI (%)	PHRR (kW/m^2^)	THR (MJ/m^2^)	TTI (s)	Tensile Strength (MPa)	Impact Strength (kJ/m^3^)	Flexural Modulus (GPa)	Ref.
TPU	425.6	19.8	769	58.4	60	-	-	-	[61]
TPU/CP-Ti_3_C_2_T_x_-0.5	428.2	21.8	783	56.2	46	-	-	-	
TPU/CP-Ti_3_C_2_T_x_-1.0	423.2	22.6	692	51.9	41	39.3	-	-	
TPU/CP-Ti_3_C_2_T_x_-2.0	425.7	22.8	433	47.6	36	-	-	-	
TPU/CP-Ti_3_C_2_T_x_-4.0	407.4	23.8	274	39.8	35	40.3	-	-	
HVP/D230	317.9	28.6	-	-	-	-	-	-	[47]
E51/D230	368.5	21.3	-	-	-	52.7	-	1.95	
HVP/D230-CFs	-	34.2	330.2	19.9	65	-	-	-	
E51/D230-CFs	-	21.1	354.7	35.3	74	213	-	40.5	
Pure BMI	426	-	589.9	63.4	-	-	6.62	-	[62]
BMI/MX@HBET 0.25	429	-	416.7	59.6	-	-	8.39	-	
BMI/MX@HBET 0.5	432	-	555.2	54.3	-	-	9.88	-	
BMI/MX@HBET 1.0	432	-	482.2	47.8	-	-	11.16	-	
BMI/MX@HBET 2.0	430	-	386.5	54.7	-	-	7.74	-	
BMI/CF	-	-	398.1	32.6	-	-	63.52	-	
BMI/CF-O-Si/MX@HBET	-	-	353.8	29.1	-	-	82.10	-	

## Data Availability

The datasets generated during the current study are available from the corresponding author on reasonable personal request.

## Abbreviations

EP	Epoxy
EPs	Epoxy resins
CF	Carbon fiber
CFs	Carbon fibers
THF	Tetrahydrofuran
HCCP	Hexachlorocyclotriphosphazene
TEA	Triethylamine
DDE	4,4′-diaminodiphenyl ether
IFSS	Interfacial shear strength
DMF	Dimethylformamide
XPS	X-ray photoelectron spectroscopy
SEM	Scanning electron microscopy
DMSO	Dimethyl sulfoxide
ILSS	Interlaminar shear strength
CFO	Oxidized carbon fibers
ODA	4,4′-oxydianiline
CF/EP	Carbon fiber/epoxy
BPS	Bis(4-hydroxyphenyl) sulfone
SN	Nucleophilic substitution
NPs	Nanoparticles
EDA	Ethanediamine
EHEP	Hexa[(4-(2,3-epoxypropyl)-2-methoxy) phenoxy]cyclotriphosphazene
APS	3-aminopropyltriethoxysilane
FTIR	Fourier transform infrared spectroscopy
PHRR	Peak heat release rate
THR	Total heat release
BMI	Bismaleimide
TSP	Total smoke production
GO	Graphene oxide
